# Predicting Consumer Adoption of Luxury Products via Instagram Marketing: A Machine Learning Approach

**DOI:** 10.12688/f1000research.169639.2

**Published:** 2025-10-23

**Authors:** Anitha Nallasivam, Gokula Krishnan S, Itam Urmila Jagadeeswari, Guruprasad Desai, Din Bandhu

**Affiliations:** 1 Department of MBA & Research Centre, Surana College, Bengaluru, Karnataka, 560060, India; 2 Department of Commerce, Manipal Academy of Higher Education, Manipal, Karnataka, India; 3 Galgotias University, Greater Noida, Uttar Pradesh, India

**Keywords:** Luxury brand adoption; Instagram marketing; Machine learning prediction, Brand trust and Consumer purchase intention

## Abstract

In the contemporary digital landscape, social media platforms have radically reshaped consumer-brand interactions, with Instagram emerging as a pivotal channel for luxury brand marketing. This study investigates the adoption of luxury products through Instagram by integrating psychological, social, and demographic predictors with advanced analytical methodologies. Utilizing a comprehensive dataset of 205 respondents, we examine how Instagram engagement metrics—such as time spent on the platform, influencer marketing, sponsored advertisements, and interactive content—impact consumer adoption behaviours. Employing multiple statistical techniques including multiple linear regression, logistic regression, and path analysis, alongside machine learning models like Support Vector Machines (SVM) and decision trees, we identify the primary drivers of purchase intention and brand trustworthiness in the luxury context. Notably, while surface-level engagement metrics show limited predictive power, factors such as brand trust, prestige, uniqueness, and personalized recommendations have strong influences on consumer purchase intention. The SVM model accurately predicts adoption likelihood with 87% accuracy, underscoring the efficacy of psychological and social variables as discriminators. Additionally, sentiment analysis of qualitative feedback reveals predominantly positive consumer attitudes toward Instagram marketing strategies. Our findings highlight the nuanced role of brand-related psychological constructs and demographic variables in shaping luxury product adoption, offering actionable insights for marketers aiming to optimize Instagram’s potential in luxury branding. This integrative approach advances both theoretical understanding and practical applications, reinforcing the importance of authenticity, emotional connection, and targeted psychographic segmentation in luxury digital marketing.

## 1. Introduction

In the digital age, social media platforms have revolutionized the landscape of consumer-brand interaction, significantly transforming marketing strategies and consumer behavior. Among these platforms, Instagram has emerged as a dominant force, especially in the context of luxury brand marketing, due to its visually rich, influencer-driven, and aspirational ecosystem ( Casaló, Flavián, & Ibáñez-Sánchez, 2020). With more than two billion monthly active users globally, Instagram serves not only as a content-sharing application but as a sophisticated visual commerce platform that enables storytelling, lifestyle marketing, and immersive brand experiences ( Statista, 2024). In particular, luxury brands have increasingly leveraged Instagram to build brand equity, stimulate emotional connections, and drive product adoption through a mix of influencer collaborations, personalized recommendations, interactive content, and curated aesthetics ( Ko, Costello, & Taylor, 2019; Lou & Yuan, 2019).

The consumer adoption of luxury products is a complex and multifaceted phenomenon, influenced not only by product-related attributes but also by psychological, social, and demographic factors. Traditional marketing models emphasize the role of brand trust, prestige, uniqueness, and loyalty in shaping purchase intentions and adoption behaviors ( Vigneron & Johnson, 2004; Chaudhuri & Holbrook, 2001). In the social media era, these constructs acquire new meaning as the digital environment enhances symbolic consumption, identity signalling, and social comparison processes, particularly among younger consumers who are digitally native and brand-conscious ( Jin & Ryu, 2020). The emergence of influencer marketing and algorithmically driven content personalization has further altered the dynamics of luxury consumption, requiring marketers to understand not only consumer preferences but also the emotional and perceptual responses elicited by digital interactions ( Phua, Jin, & Kim, 2020).

Despite the growing body of literature on luxury branding and digital marketing, significant research gaps remain, particularly regarding the integration of data-driven approaches such as machine learning (ML) with behavioral theories to understand and predict consumer adoption. While previous studies have explored isolated factors influencing purchase intention, few have developed comprehensive predictive models that simultaneously incorporate engagement metrics, psychological variables, content typologies, and demographics within a social media marketing context ( Ko et al., 2019; Kim & Kim, 2021). Furthermore, limited empirical attention has been paid to sentiment analysis as a method to decode consumer emotions and perceptions from open-ended feedback—an area with high potential to inform marketing strategies and personalize user experiences ( Pang & Lee, 2008).

### 1.1 Objectives of the study

a) To assess the impact of Instagram engagement metrics on consumer adoption of luxury products.
b) To identify key psychological and brand factors influencing purchase intention.
c) To analyze the relationship between social media content types and brand trustworthiness.
d) To develop and validate machine learning models for predicting consumer adoption likelihood.
e) To evaluate demographic influences on consumer adoption behavior.

This research addresses the study objectives by adopting a mixed-methods approach, integrating quantitative analysis (via machine learning and statistical modeling) with qualitative insights (through sentiment analysis) to examine the determinants of consumer adoption of luxury products on Instagram. The study is grounded in robust theoretical frameworks, including the Theory of Planned Behavior ( Ajzen, 1991), Social Identity Theory ( Tajfel & Turner, 1986), and the Elaboration Likelihood Model, which collectively emphasize the roles of attitudinal, identity-based, and emotional variables in shaping consumer decision-making. These frameworks provide a conceptual foundation for modeling how engagement (e.g., time spent on Instagram, interaction with influencer content), brand-related perceptions (e.g., trust, prestige, uniqueness), and demographic attributes (e.g., age, income, education) influence consumer adoption behaviors in a luxury context.

This study aims to bridge the theoretical and empirical gaps in understanding consumer adoption of luxury products via Instagram by: (1) identifying the key psychological, social, and demographic predictors of adoption; (2) evaluating the impact of content types and engagement on brand trustworthiness and purchase intention; (3) leveraging machine learning models to forecast adoption behavior; and (4) extracting emotional and thematic insights from qualitative consumer feedback. Through this integrative lens, the research not only advances academic discourse but also provides actionable insights for practitioners in luxury branding and digital marketing.

## 2. Review of literature and development of hypothesis

H1: Instagram engagement is significantly associated with consumer adoption of luxury products

The increasing prominence of Instagram as a marketing tool has significantly reshaped how luxury brands interact with consumers. With over two billion monthly active users globally, Instagram has evolved from a mere photo-sharing application to a robust platform for brand storytelling, influencer collaboration, and customer engagement ( Statista, 2024). Among the many marketing domains impacted, luxury branding has seen some of the most pronounced transformations, particularly due to the visual nature of Instagram that allows the projection of aspirational lifestyles—an essential characteristic of luxury brand identity ( Kapferer & Bastien, 2012). Consumer engagement on Instagram is multidimensional, encompassing time spent on the platform, interaction with influencer-generated content, responsiveness to story and sponsored ads, and participation in interactive elements like polls or contests ( Casaló, Flavián, & Ibáñez-Sánchez, 2020). This engagement forms a new kind of digital customer journey where visibility, relatability, and emotional resonance replace traditional advertising techniques. Jin and Ryu (2020) argue that Instagram influencers often function as micro-celebrities, building parasocial relationships with followers that can translate into high perceived trust and purchasing intent.

The theory of reasoned action ( Ajzen & Fishbein, 1980) and the elaboration likelihood model provide strong theoretical bases for this relationship. According to these theories, consumers exposed to persuasive content—such as influencer endorsements and branded storytelling—develop more favorable attitudes toward the product and, ultimately, increased intention to adopt. Influencer credibility, message relevance, and emotional appeal are proven to moderate this pathway ( Lou & Yuan, 2019). A study by De Veirman, Cauberghe, and Hudders (2017) highlights that influencers with a high follower-to-engagement ratio generate more trust, which in turn predicts consumer adoption behavior in luxury segments. Moreover, Instagram’s interactive features, such as “swipe-up” links, polls, Q&A stickers, and immersive advertisements, further drive adoption by reducing the psychological distance between consumer and brand. These functionalities create a pseudo-involvement effect, allowing consumers to feel more personally connected to brand narratives ( Phua, Jin, & Kim, 2020). In luxury product marketing, where experiential appeal and brand symbolism are critical, this personal engagement is especially influential.

Empirical studies corroborate the strong link between Instagram engagement and actual purchase behavior. For instance, a quantitative study by Djafarova and Trofimenko (2019) on millennials’ luxury consumption through Instagram revealed that higher interaction frequency significantly predicts purchase intention and product adoption. Similarly, Kim and Kim (2021) found that consumers who engage more with brand content on Instagram report higher levels of emotional connection and are more likely to purchase high-value products. Taken together, these findings suggest a substantive and statistically relevant relationship between Instagram engagement and luxury product adoption. Thus, based on social influence theory, digital consumer behavior literature, and empirical research: *H1: Instagram engagement is significantly associated with consumer adoption of luxury products.*

H2: Brand trust, prestige, uniqueness, and personalized recommendations significant predictors of consumer purchase intentions for luxury products marketed on Instagram

In the context of luxury marketing, purchase intention is heavily influenced by several brand-related attributes—primarily trust, prestige, uniqueness, and the ability to provide personalized experiences. As social media platforms increasingly serve as the main interface between luxury brands and consumers, these dimensions have become even more vital in shaping consumer attitudes and behavioral intentions ( Ko, Costello, & Taylor, 2019). Instagram, in particular, amplifies these characteristics by allowing brands to craft visually compelling narratives, showcase exclusivity, and tailor content to specific segments through algorithmic recommendations and influencer collaborations. Brand trust is a fundamental component of purchase decision-making, especially in digital environments where the tangibility of the product and physical brand interactions are absent. Trust facilitates the perceived reliability and integrity of a brand, which is crucial in the high-risk context of luxury purchases ( Chaudhuri & Holbrook, 2001). Bian and Forsythe (2012) found that online luxury shoppers heavily rely on brand trust as a proxy for product authenticity and value. On Instagram, brand trust is often fostered through consistent messaging, influencer partnerships, transparency in sponsored content, and responsiveness to consumer queries.

Brand prestige—defined as the perceived status and social value associated with a brand—also plays a critical role. Luxury brands often derive their prestige from heritage, craftsmanship, and celebrity associations ( Vigneron & Johnson, 2004). On Instagram, these elements are manifested through curated feeds, exclusive launch announcements, and endorsements by high-profile influencers. Such tactics align with symbolic interaction theory, which suggests that consumers derive identity and status from the symbolic meanings of possessions ( Solomon, 1983). Brand uniqueness is another essential determinant of luxury brand appeal. Consumers of luxury products often seek differentiation from the mainstream market, and this pursuit of uniqueness significantly affects their intention to purchase ( Tian, Bearden, & Hunter, 2001). Instagram enables brands to communicate this uniqueness through limited edition product teasers, bespoke styling tips, and aesthetic brand storytelling. Research indicates that consumers perceive such uniqueness as enhancing personal identity and self-expression, thereby driving purchase motivation ( Ko et al., 2019).

Personalized recommendations, often enabled by machine learning algorithms on platforms like Instagram, have increasingly become influential in converting browsing behavior into actual purchase intention. According to Bleier, De Keyser, and Verleye (2018), personalization in online environments improves perceived relevance, satisfaction, and emotional attachment, all of which enhance purchase intent. For luxury brands, offering personalized product suggestions—based on previous interactions or demographic profiling—signals attentiveness and exclusivity, core values of luxury branding. Combining these factors, several theoretical frameworks offer insight into their joint influence on purchase intention. The Theory of Planned Behavior ( Ajzen, 1991) suggests that attitudes toward behavior (driven by trust, prestige, uniqueness) and perceived behavioral control (enhanced by personalization) are central to forming purchase intentions. The brand equity model also supports this, indicating that strong brand associations and positive experiences build consumer preference and drive buying behavior ( Aaker, 1991).

Empirical studies provide robust support for these relationships. Ko et al. (2019) confirmed that brand prestige, trust, and uniqueness significantly predict intention to purchase luxury products. Similarly, Tuten and Solomon (2020) reported that personalized content delivery via Instagram improves click-through rates and conversion in luxury e-commerce. Thus, synthesizing insights from branding theory, digital marketing literature, and consumer psychology, it is hypothesized that: *H2: Brand trust, prestige, uniqueness, and personalized recommendations significant predictors of consumer purchase intentions for luxury products marketed on Instagram.*

H3: The type of social media content shared on Instagram is a significant predictor of consumers perception of brand trustworthiness

Brand trustworthiness is a pivotal construct in consumer behavior, especially in the digital marketplace where trust substitutes for the absence of physical interaction ( Chaudhuri & Holbrook, 2001). On Instagram—a platform that thrives on aesthetics, narrative, and engagement—the nature and quality of content play a significant role in shaping how trustworthy a brand is perceived to be ( Casaló, Flavián, & Ibáñez-Sánchez, 2018). The growing reliance on social media as a primary source of information and inspiration in the luxury domain underscores the importance of understanding how various content types influence consumers’ perception of brand credibility and reliability. Research suggests that user-generated content (UGC) significantly contributes to perceived brand trustworthiness, particularly due to its authenticity and relatability. UGC includes reviews, testimonials, tagged photos, and influencer collaborations not directly orchestrated by the brand. According to Christodoulides, Jevons, and Bonhomme (2012), such content reduces psychological distance and enhances consumer identification with the brand, thereby fostering trust. Consumers often view content shared by fellow users as more credible than professionally produced advertisements, particularly in the luxury domain where authenticity reinforces exclusivity and social proof ( Pentina, Zhang, & Basmanova, 2013).

In parallel, brand storytelling on Instagram—via curated narratives, heritage posts, behind-the-scenes features, and visual campaigns—helps construct a consistent brand image that aligns with consumer values and aspirations. Escalas (2004) posits that narrative-based advertising facilitates emotional connections, enabling consumers to integrate brand stories into their personal identity. For luxury brands, storytelling may revolve around craftsmanship, legacy, and exclusivity, thereby reinforcing brand ethos and establishing trust ( Kapferer & Bastien, 2012). Another key form of content that affects brand trustworthiness is product showcases, which offer detailed insights into product attributes, usage, and styling. These visuals serve as evidence of quality, consistency, and transparency—key antecedents of trust ( Keller, 2009). On Instagram, high-resolution product photography, carousel posts, and demonstration videos not only highlight functional aspects but also signal competence and attention to detail, strengthening brand credibility.

Additionally, interactive content—such as polls, quizzes, swipe-up links, live sessions, and Q&As—fosters a two-way communication model that promotes consumer involvement and engagement. According to Malthouse and Calder (2011), this interaction builds relational trust as consumers perceive the brand to be more responsive, transparent, and interested in consumer feedback. For luxury brands, interactivity can convey a sense of exclusivity and personalized attention, thereby enhancing trustworthiness. The Media Richness Theory ( Daft & Lengel, 1986) and Source Credibility Theory ( Hovland & Weiss, 1951) provide theoretical grounding for the impact of content types on trust. Richer, interactive, and user-driven content is perceived as more reliable and credible than passive, one-directional communication. Empirical studies echo this; Casaló et al. (2020) found that social media content richness positively affects brand trust and loyalty intentions. In the context of Instagram, where visual, interactive, and community-driven content coexist, each type plays a differentiated but synergistic role in shaping consumer trust. Thus, drawing upon digital branding, social psychology, and empirical research: *H3: The type of social media content shared on Instagram is a significant predictor of consumers perception of brand trustworthiness.*

H4: Psychological and Social Factors Significantly Predict the Likelihood of Consumer Adoption of Luxury Products

The adoption of luxury products, particularly in a digital marketing context like Instagram, is influenced by a confluence of psychological and social factors that shape consumer attitudes, behaviors, and decision-making processes. These factors include brand loyalty, brand trust, perceived prestige, and brand uniqueness—constructs that are closely associated with emotional, symbolic, and identity-based motivations for luxury consumption ( Kapferer & Bastien, 2012).

Brand loyalty is a key psychological factor that influences repeat purchasing and advocacy behavior. In the luxury sector, loyalty is not merely transactional but deeply emotional and identity-driven. Consumers often form long-standing relationships with luxury brands that resonate with their personal values and aspirations ( Chaudhuri & Holbrook, 2001). On Instagram, brand loyalty is reinforced through consistent visual narratives, influencer endorsements, and direct engagement with followers. Oliver (1999) argues that true brand loyalty entails a deeply held commitment to re-purchase despite situational influences and marketing efforts by competitors—making it a strong predictor of future adoption behavior.

Brand trust is another critical psychological dimension, particularly relevant in the online luxury marketplace where consumers must rely on symbolic cues, visual content, and third-party endorsements rather than tactile experience. Trust reduces perceived risk and increases confidence in the quality, authenticity, and status value of the product ( Bian & Forsythe, 2012). Trust in luxury brands is often fostered through transparent communication, ethical practices, and credible influencer collaborations on platforms like Instagram ( Casaló, Flavián, & Ibáñez-Sánchez, 2020).

Perceived brand prestige functions as a social signaling mechanism in luxury consumption. According to social identity theory ( Tajfel & Turner, 1986), individuals derive a sense of belonging and self-esteem from the social groups they affiliate with, and luxury brands serve as tools for this identity construction. Vigneron and Johnson (2004) suggest that consumers value luxury products for their ability to confer prestige, which is often enhanced through Instagram’s visual storytelling capabilities, celebrity endorsements, and exclusive product launches.

Brand uniqueness, defined as the perception of a brand as being distinct and not easily substitutable, also significantly influences adoption likelihood. Consumers of luxury products often seek distinctiveness as a way to express individuality and elevate social status ( Tian, Bearden, & Hunter, 2001). Instagram enhances this perception by allowing brands to create tailored experiences, limited-time offers, and curated content that emphasize exclusivity and innovation.

From a theoretical perspective, the Theory of Consumption Values ( Sheth, Newman, & Gross, 1991) explains that consumers evaluate products based on functional, emotional, and social values. Each of the constructs outlined—trust, loyalty, prestige, and uniqueness—contributes to these evaluative dimensions, affecting the perceived utility and desirability of the brand, and therefore, the likelihood of its adoption. In addition, the Self-Congruity Theory ( Sirgy, 1982) posits that individuals are more likely to adopt brands that reflect their self-image or ideal self, reinforcing the role of psychological alignment in luxury consumption. Empirical studies validate these relationships. For instance, Ko et al. (2019) found that brand prestige and uniqueness significantly predict consumer intention to adopt luxury brands in digital contexts. Similarly, research by Hung, Chen, and Peng (2013) revealed that brand trust and emotional attachment are major drivers of luxury brand preference and loyalty. Hence, considering the convergence of social psychology, digital consumer behavior, and luxury marketing literature: *H4: Psychological and social factors, including brand loyalty, trust, prestige, and uniqueness, significantly predict the likelihood of consumer adoption of luxury products.*

H5: Demographic factors have a significant association with the adoption of luxury products marketed through Instagram

Demographic factors such as age, gender, income level, and education have long been recognized as critical variables influencing consumer behavior, particularly in the context of luxury consumption. With the proliferation of digital marketing and the rise of Instagram as a dominant platform for luxury branding, it becomes essential to reassess how these demographic variables affect the adoption of luxury products in a social media context. The dynamics of digital interaction, visual engagement, and socio-technical fluency vary widely across demographic groups, shaping both the perception of luxury and actual purchasing behavior ( Ladhari, Gonthier, & Lajante, 2019). Age is one of the most significant demographic variables, particularly in digital contexts. Millennials and Gen Z consumers represent the primary Instagram user base and exhibit higher engagement with visual and interactive content ( Pew Research Center, 2023). These younger cohorts are more receptive to influencer marketing, user-generated content, and personalized experiences—elements commonly used by luxury brands on Instagram. According to Kim and Kim (2021), younger consumers are not only more digitally literate but also associate luxury with lifestyle alignment and social currency, which makes them more susceptible to Instagram-driven brand narratives. Conversely, older consumers may exhibit lower levels of adoption due to platform unfamiliarity or differing luxury value perceptions.

Gender also plays a vital role in shaping luxury adoption. Numerous studies have highlighted that women tend to engage more with luxury fashion and cosmetic products, driven by hedonic and symbolic motivations ( Jin & Ryu, 2020). On Instagram, gender differences manifest in content preferences, influencer followership, and emotional responsiveness to visual marketing. For instance, women are more influenced by aesthetic product showcases and emotionally rich storytelling, whereas men may respond more to technical and exclusivity-related cues ( Khare & Rakesh, 2011). Such variances imply that gender moderates both engagement and adoption patterns in luxury consumption. Income level is perhaps the most intuitive demographic predictor of luxury product adoption. Luxury consumption traditionally aligns with higher disposable income, but digital platforms like Instagram are reshaping this narrative by making luxury “aspirational” rather than “attainable” ( Kapferer & Bastien, 2012). This has given rise to a new segment of aspirational consumers—those who may not yet have the means but engage with luxury content for social identity signaling ( Truong, Simmons, McColl, & Kitchen, 2008). While high-income individuals still dominate actual purchase behavior, the influence of Instagram is expanding the scope of luxury branding to include symbolic interaction with aspirational consumers, making income a nuanced moderator.

Education level affects consumer literacy, product discernment, and susceptibility to marketing influence. Educated consumers often seek more information-rich and authentic content before making high-involvement purchases like luxury goods. They may be more critical of influencer endorsements and more sensitive to ethical branding practices, such as sustainability and fair trade ( Ko et al., 2019). Additionally, individuals with higher education are more likely to align their consumption with self-identity and social status, concepts deeply embedded in luxury branding.

From a theoretical standpoint, demographic segmentation draws upon Maslow’s hierarchy of needs ( Maslow, 1943), where luxury purchases typically satisfy esteem and self-actualization needs. Moreover, diffusion of innovations theory ( Rogers, 2003) suggests that early adopters (often younger, educated, and higher-income individuals) are more receptive to innovations such as social media luxury marketing. Empirical studies support these relationships. Ladhari et al. (2019) found that demographic variables significantly impact luxury purchase motivations and attitudes on digital platforms. Similarly, Dubois and Laurent (1994) emphasized that income and education predict both willingness and ability to purchase luxury goods. Therefore, grounded in consumer behavior theory and supported by empirical evidence: *H5: Demographic factors have a significant association with the adoption of luxury products marketed through Instagram.*

H6: Positive Consumer Sentiment Toward Instagram-Based Marketing of Luxury Products Is Significantly Associated With Higher Consumer Adoption

In the evolving digital landscape, consumer sentiment—the emotional tone, attitude, and evaluative response toward marketing communications—has become a critical determinant of consumer behavior, especially in luxury brand marketing via social media platforms like Instagram. Instagram’s highly visual and immersive environment offers an emotionally charged medium where luxury brands cultivate aspirational appeal, narrative depth, and interactive intimacy. Within this context, positive consumer sentiment has a substantial influence on consumer adoption behavior, often mediating the relationship between exposure to brand content and actual purchasing decisions ( Pang & Lee, 2008). The concept of sentiment has deep roots in affect theory, which emphasizes the role of emotional states in shaping perception, judgment, and behavior ( Bagozzi, Gopinath, & Nyer, 1999). Emotional responses to Instagram content—such as admiration, desire, trust, or even envy—are known to trigger psychological processes such as identification, self-congruence, and social comparison, all of which are highly relevant in luxury consumption ( Ko, Costello, & Taylor, 2019). When consumers react positively to branded Instagram content, whether through likes, comments, shares, or sentimentally rich responses, they are more likely to convert from passive followers to active adopters of luxury products.

Sentiment analysis, a technique used in computational linguistics and machine learning, enables the extraction of emotional tone from text data (e.g., comments, captions, hashtags). Studies show that sentiment polarity (positive vs. negative) and intensity significantly correlate with engagement metrics and behavioral outcomes ( Thelwall et al., 2010). In luxury marketing, a stream of positive sentiment around influencer campaigns, product launches, or user-generated content reinforces brand equity and enhances perceived desirability ( Gensler, Völckner, Liu-Thompkins, & Wiertz, 2013). The Elaboration Likelihood Model (ELM) provides theoretical grounding for how sentiment operates. In the peripheral route to persuasion, which is particularly dominant on platforms like Instagram, emotional cues and affective responses take precedence over rational evaluations. Aesthetic appeal, influencer charisma, and positive emotional valence become sufficient to elicit favorable attitudes and intentions. Therefore, if consumers experience positive affect in response to Instagram marketing efforts, the likelihood of subsequent adoption increases.

In the context of luxury products—where the purchase is not driven by utilitarian value but by status signaling, self-expression, and hedonic pleasure—the importance of emotional alignment becomes even more pronounced. Positive sentiment suggests that the consumer not only accepts the brand’s message but aligns with its aspirational promise and symbolic identity. According to Phua, Jin, and Kim (2020), emotional bonding through social media marketing improves brand love, which in turn increases purchase likelihood, especially in fashion and luxury domains. Empirical studies support this view. Kim and Ko (2012) demonstrated that emotional engagement with luxury brand posts on social media increases brand value and purchase intention. Similarly, a study by Hudson, Huang, Roth, and Madden (2016) found that emotional tone in social media storytelling significantly influenced consumer-brand relationships and behavioral intentions. Thus, supported by affective psychology, digital marketing theory, and empirical findings: *H6: Positive consumer sentiment toward Instagram-based marketing of luxury products is significantly associated with higher consumer adoption.*

### 2.1 Theoretical Foundations and Conceptual Model

The conceptualization of consumer adoption of luxury products via Instagram is grounded in several foundational behavioral theories, each articulating distinct drivers and underlying mechanisms in the digital landscape. The Theory of Planned Behavior (TPB) posits that attitudes toward behavior, perceived social norms, and behavioral control collectively shape individuals’ intentions and subsequent actions ( Ajzen, 1991). Within the context of luxury marketing on Instagram, this framework elucidates how brand trust, prestige, uniqueness, and personalized engagement inform favorable attitudes and purchase intentions.

The Elaboration Likelihood Model (ELM) proposed by Petty and Cacioppo (1986) further delineates the dual pathways—central (rational) and peripheral (emotional or heuristic)—through which persuasion occurs. Most luxury marketing on Instagram primarily activates the peripheral route, leveraging influencer charisma, compelling visual storytelling, and emotional resonance to stimulate product adoption, particularly when cognitive engagement is minimal.

Social Identity and Self-Congruity Theories emphasize that consumers gravitate toward brands and experiences aligned with their social identities and aspirational selves ( Tajfel & Turner, 1986; Sirgy, 1982). Instagram’s visual-social medium significantly enhances consumers’ opportunities for self-expression, status signaling, and symbolic distinction through luxury product adoption. Similarly, the Stimulus-Organism-Response (S-O-R) Model explains how social media stimuli—like interactive brand content, entertainment, personalization, and electronic Word-of-Mouth—impact internal consumer states, resulting in cognitive, affective, and behavioral engagement with luxury brands ( Mehrabian, 1974; Cheung & To, 2021).

Empirical literature reinforces these theoretical orientations. As detailed in the preceding review, robust associations exist between Instagram engagement and luxury product adoption; critical brand factors such as trust, prestige, and uniqueness enhance purchase intentions; content type directly affects perceptions of brand trustworthiness; and demographic characteristics and consumer sentiment function as pivotal moderators and predictors in these relationships.

### 2.2 Key Constructs and Relationships – proposed conceptual framework

**Figure 1.**
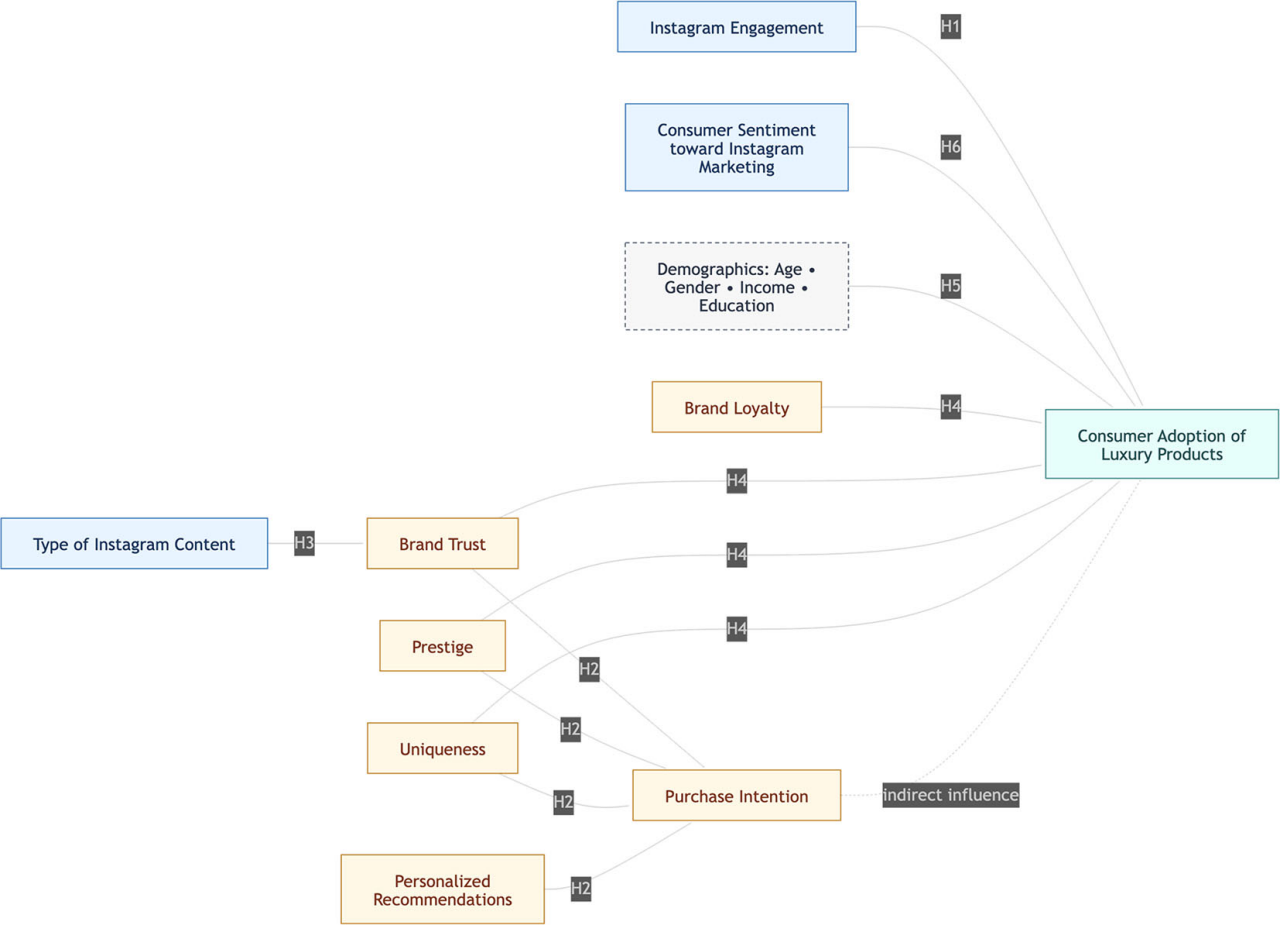
Proposed conceptual framework.

Based on the theoretical bases and literature review, the following main constructs and linkages frame the study as shown in the Figure 1.

Instagram engagement is multidimensional, encompassing time spent on the platform, interactions with influencers and brands, responsiveness to sponsored and story ads, and participation in interactive features such as polls and Q&As. Critical brand factors like trust, prestige, uniqueness, and personalized recommendations all shape consumers’ purchase intentions and adoption likelihood. Brand content on Instagram can be categorized as influencer- or brand-created, user-generated, narrative-driven, or interactive—each type having distinct effects on perceptions of trustworthiness. Psychological and social drivers, including loyalty, emotional attachment, social identity, desire for uniqueness, and prestige, further fuel the adoption of luxury products. Demographic characteristics such as age, gender, education, and income act as moderators, influencing how Instagram engagement and brand attributes translate into adoption decisions. Notably, consumer sentiment—the prevailing emotional tone toward Instagram marketing—serves as a key mediating factor, shaping the nature and strength of relationships between these variables and actual luxury product adoption.

The integrated theoretical and conceptual model framed in this study provides a robust foundation for investigating how Instagram engagement, brand-related factors, content types, psychological and social drivers, demographics, and consumer sentiment collectively influence the adoption of luxury products. Grounded in multidimensional behavioral theories and reinforced by empirical evidence, the proposed framework identifies both direct and indirect relationships. This conceptual base directly informs the subsequent methodological approach, guiding the selection of analytical techniques—such as regression analysis to empirically validate the hypothesized linkages and advance actionable insights for both academic research and luxury brand strategists in the digital domain.

## 3. Methodology

This study adopts a mixed-methods research design, integrating both quantitative and qualitative approaches to examine the determinants of consumer adoption of luxury products through Instagram marketing. The methodological approach was chosen to enhance both predictive rigor and contextual depth, ensuring a holistic understanding of consumer behavior in digital luxury environments. Primary data were collected from a purposive sample of 205 respondents via a structured questionnaire that included Likert-scale items measuring Instagram engagement, brand-related psychological constructs (trust, prestige, uniqueness, loyalty), and consumer adoption variables, along with demographic questions (age, gender, income, and education). For the purpose of this study, ‘luxury products’ were explicitly defined to respondents as high-involvement, premium-priced goods from non-essential categories where brand identity, craftsmanship, and symbolic value are primary purchase drivers ( Kapferer & Bastien, 2012). The survey instructions specified that respondents should base their answers on categories such as luxury fashion (e.g., handbags, apparel from brands like Gucci or Louis Vuitton), premium accessories (e.g., watches, jewellery), high-end cosmetics, and designer footwear. Open-ended questions were also incorporated to capture qualitative insights on consumer sentiment. The questionnaire was shared online using Google Forms and disseminated across India, targeting consumers who access Instagram for luxury product purchases. The instrument was pre-tested for reliability and content validity, and the final dataset was analyzed using both descriptive and inferential techniques. Descriptive statistics were employed to profile the respondent demographics and digital behavior patterns. To assess the influence of key variables on consumer adoption, a range of advanced statistical and machine learning techniques were applied. Multiple Linear Regression was used to evaluate the effect of Instagram engagement metrics on luxury product adoption, while Logistic Regression examined the role of brand trust, prestige, and uniqueness on purchase intention. Path Analysis was conducted to model the structural relationships between different types of social media content and brand trustworthiness. In addition, Support Vector Machines (SVM) and Decision Tree algorithms were employed to predict consumer adoption likelihood based on psychological and demographic features, offering robustness against non-linear relationships and enhancing predictive performance. Visual analytics were integral to the interpretative strategy, with Parallel Coordinates Plots used to display multidimensional engagement data, Radar Charts for visualizing factor importance in influencing purchase intention, Heatmaps to compare predicted versus actual adoption patterns, and Sentiment Distribution Graphs to interpret emotional tone in qualitative feedback. Furthermore, sentiment analysis of open-ended responses was conducted using lexicon-based and machine learning methods (e.g., VADER and TextBlob), enabling thematic exploration of emotional drivers such as trust in influencers, admiration for brand prestige, desire for exclusivity, and skepticism toward over-commercialized promotions. This combination of predictive modeling and qualitative sentiment analysis provides an integrative framework that captures both the measurable and affective dimensions of consumer behavior. By aligning statistical precision with narrative insight, the methodology offers a comprehensive lens to understand how Instagram marketing strategies influence luxury product adoption, and contributes to methodological advancements in digital consumer research, luxury branding, and applied machine learning in marketing contexts.

This study was conducted in accordance with the principles of the Declaration of Helsinki. Ethical review and approval have been obtained from the The Institutional Review Board (IRB) of Galgotias University, Greater Noida, Uttar Pradesh, India. In compliance with the ICMR National Ethical Guidelines for Biomedical and Health Research Involving Human Participants (2017), the study was classified as minimal-risk research. It employed anonymous surveys with adult participants on non-sensitive topics (consumer behavior and marketing perceptions), and no identifiable information was collected. Verbal informed consent was obtained from all participants prior to their participation. Verbal consent was chosen over written consent to guarantee complete participant anonymity, as collecting signed consent forms would have required the recording of identifying information.

## 4. Data analysis

The data analysis phase of this study employed a combination of statistical and machine learning techniques to comprehensively explore the factors associated with consumer adoption of luxury products through Instagram marketing. Initial descriptive statistics provided an overview of key demographic characteristics and consumer behavior metrics. Multiple linear and logistic regression models were applied to examine the predictive relationships between Instagram engagement, brand-related psychological constructs, and purchase intentions. Path analysis was conducted to estimate the associations between various social media content types and brand trustworthiness. To enhance predictive accuracy, Support Vector Machine (SVM) models were utilized to forecast consumer adoption likelihood based on psychological and social factors. Additionally, decision tree models provided interpretable patterns in demographic associations with consumer behavior. Finally, sentiment analysis was performed on qualitative feedback to capture emotional responses to Instagram marketing strategies. This multifaceted analytical approach provided comprehensive insight into both quantitative and qualitative dimensions associated with luxury product adoption on Instagram.

The descriptive analysis presented in Table 1 provides an overview of the key demographic and behavioral variables within the sample, including age, gender, income level, education, time spent on Instagram, and consumer adoption levels. These foundational statistics set the stage for deeper analysis by characterizing the participant profile and their Instagram usage patterns. Building on this, Table 2 presents the results of a multiple linear regression analysis examining the relationship between various Instagram engagement dimensions—such as time spent on the platform, influencer marketing, sponsored ads, story ads, and interactive content—and consumer adoption of luxury products. While the model’s intercept indicates a significant baseline level of adoption, none of the engagement factors showed statistically significant predictive value, highlighting a disconnect between superficial engagement metrics and actual adoption behavior in the luxury context. This finding suggests that other psychological or brand-related constructs may be more predictive of consumer purchase decisions.

**Table 1.** Descriptive statistics of demographic and behavioral variables.

Variable	Count	Mean	Std Dev	Min	25%	50%	75%	Max
Age	205	2.4	0.66	1	2	2	3	3
Gender	205	2.02	0.82	1	1	2	3	3
Income Level	205	1.86	0.82	1	1	2	3	3
Education	205	2.36	1.16	1	1	2	3	4
Time Spent on Instagram (mins)	205	2.4	1.17	1	1	2	4	4
Consumer Adoption	205	2.8	1.36	1	2	3	4	5

**Table 2.** Multiple linear regression results: Relationship between instagram engagement and consumer adoption.

Objective	Dependent variable	Independent variables	Suggested technique	New kind of chart
1. Assess the relationship between Instagram engagement and consumer adoption of luxury products	Consumer Adoption	Time Spent on Instagram, Influencer Marketing, Sponsored Ads, Story Ads, and Interactive Content	Multiple Linear Regression	Parallel Coordinates Plot

The purpose of the following analyse is to examine the influence of engagement on Instagram towards consumer adoption of luxury products as determined by factors including Instagram time, influencer advertisements, sponsored, story advertisements, and interactive advertisements. The drivers are identified by employing multiple linear regression as well as random forest algorithms, while behavior of the consumer is traced on these factors by employing a parallel coordinates plot.

Table 2 presents the results of a multiple linear regression analysis conducted to examine the influence of various dimensions of Instagram engagement—specifically, time spent on the platform, influencer marketing, sponsored advertisements, story ads, and interactive content—on the consumer adoption of luxury products. The model’s intercept is statistically significant ( *β* = 3.3147, *p* < .001), indicating a baseline level of consumer adoption that is independent of the included predictors. However, none of the independent variables exhibit statistically significant relationships with the dependent variable, as indicated by *p*-values well above the conventional threshold of 0.05.

Table 3 presents the coefficient for Time Spent on Instagram, which is negative ( *β* = -0.0115, *p* = .889), indicating that increased time spent on the platform does not significantly predict consumer adoption of luxury products. This finding challenges the assumption that prolonged exposure inherently leads to greater adoption behavior. Similar trends are observed for Influencer Marketing ( *β* = 0.0163, *p* = .806), Sponsored Ads ( *β* = -0.0995, *p* = .147), Story Ads ( *β* = -0.0333, *p* = .612), and Interactive Content ( *β* = -0.0495, *p* = .466), none of which show statistically significant associations with luxury product adoption. These results suggest a disconnect between surface-level Instagram engagement metrics and actual consumer behavior in the luxury context. Although influencer marketing and digital advertising are commonly employed strategies, their limited significance in this model suggests that mere exposure or interaction may be insufficient to trigger adoption decisions. This supports previous studies that assert emotional connection, content authenticity, and value congruence are stronger predictors of consumer behavior in luxury markets ( Ko, Costello, & Taylor, 2019; Hudson et al., 2016).

**Table 3.** Regression coefficients for instagram engagement variables.

Variable	Coef	std err	T	P>|t|	[0.025	0.975]
const	3.3147	0.441	7.518	0	2.445	4.184
Time Spent on Instagram (mins)	-0.0115	0.082	-0.14	0.889	-0.173	0.15
Influencer Marketing	0.0163	0.066	0.245	0.806	-0.115	0.147
Sponsored Ads	-0.0995	0.068	-1.455	0.147	-0.234	0.035
Story Ads	-0.0333	0.066	-0.508	0.612	-0.163	0.096
Interactive Content	-0.0495	0.068	-0.73	0.466	-0.183	0.084

One possible explanation lies in the sophisticated nature of luxury consumers, who often engage with brands based on deeper psychological, cultural, and symbolic factors rather than overt marketing tactics. As Vigneron and Johnson (2004) emphasize, luxury brand consumption is primarily driven by prestige, uniqueness, and status signaling. When social media content lacks these qualities or appears overly commercialized, it may fail to resonate with the target audience ( Djafarova & Trofimenko, 2019).

**Figure 2.**
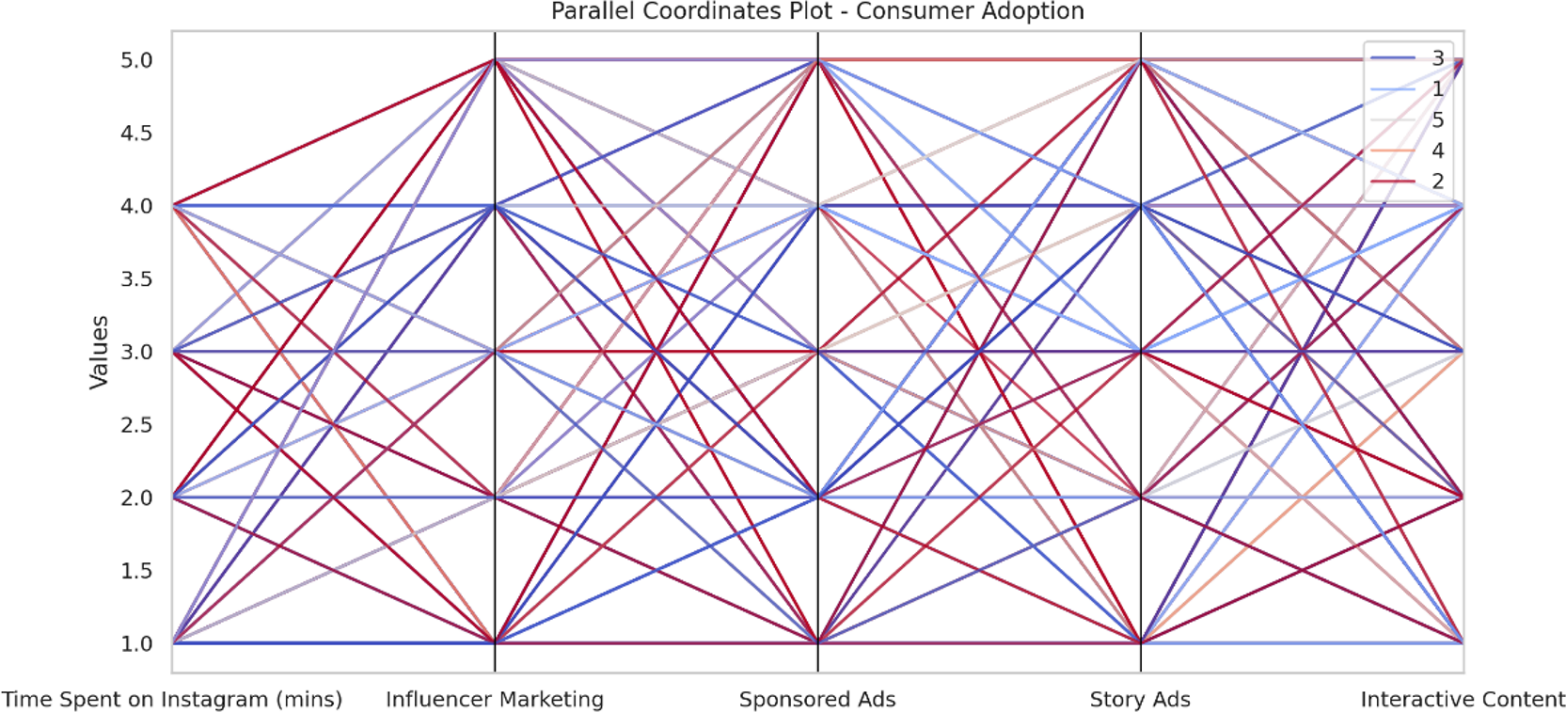
Parallel coordinates plot.

The analysis of Instagram engagement dimensions on consumer adoption was further illustrated through a Parallel Coordinates Plot, which visually depicts the multivariate relationships and patterns spent across time on Instagram, influencer marketing, sponsored ads, story ads, and interactive content (see Figure 2). This visualization complements the regression results by revealing the complex interplay and variation within the engagement metrics, even though none individually showed statistically significant predictive power in the linear model.

Furthermore, the results align with the perspective of consumer skepticism toward influencer-endorsed or sponsored content. With increasing awareness of paid collaborations, consumers may view such content as less authentic or credible ( Audrezet, de Kerviler, & Moulard, 2020). This underscores the need for luxury brands to develop more personalized, emotionally engaging, and community-driven content strategies, rather than relying solely on traditional influencer or advertising formats. The statistical insignificance of these predictors also highlights the potential limitations of traditional marketing metrics in capturing behavioral nuance and affective responses. As Phua, Jin, and Kim (2020) and Lou and Yuan (2019) suggest, factors such as emotional resonance, narrative immersion, and user-generated content credibility may offer better explanatory power when modeling consumer behavior in digital luxury contexts.

While the current regression model provides a useful preliminary analysis, the lack of significant findings implies that luxury consumer adoption via Instagram is driven by more complex, intangible factors not captured by the present variables. Future studies should integrate emotional, cognitive, and relational constructs, including perceived authenticity, self-congruity, and parasocial interaction, to better understand and predict adoption behavior. Additionally, qualitative methods such as sentiment analysis and in-depth interviews may offer richer insights into the motivations and expectations of digitally engaged luxury consumers.

**Figure 3.**
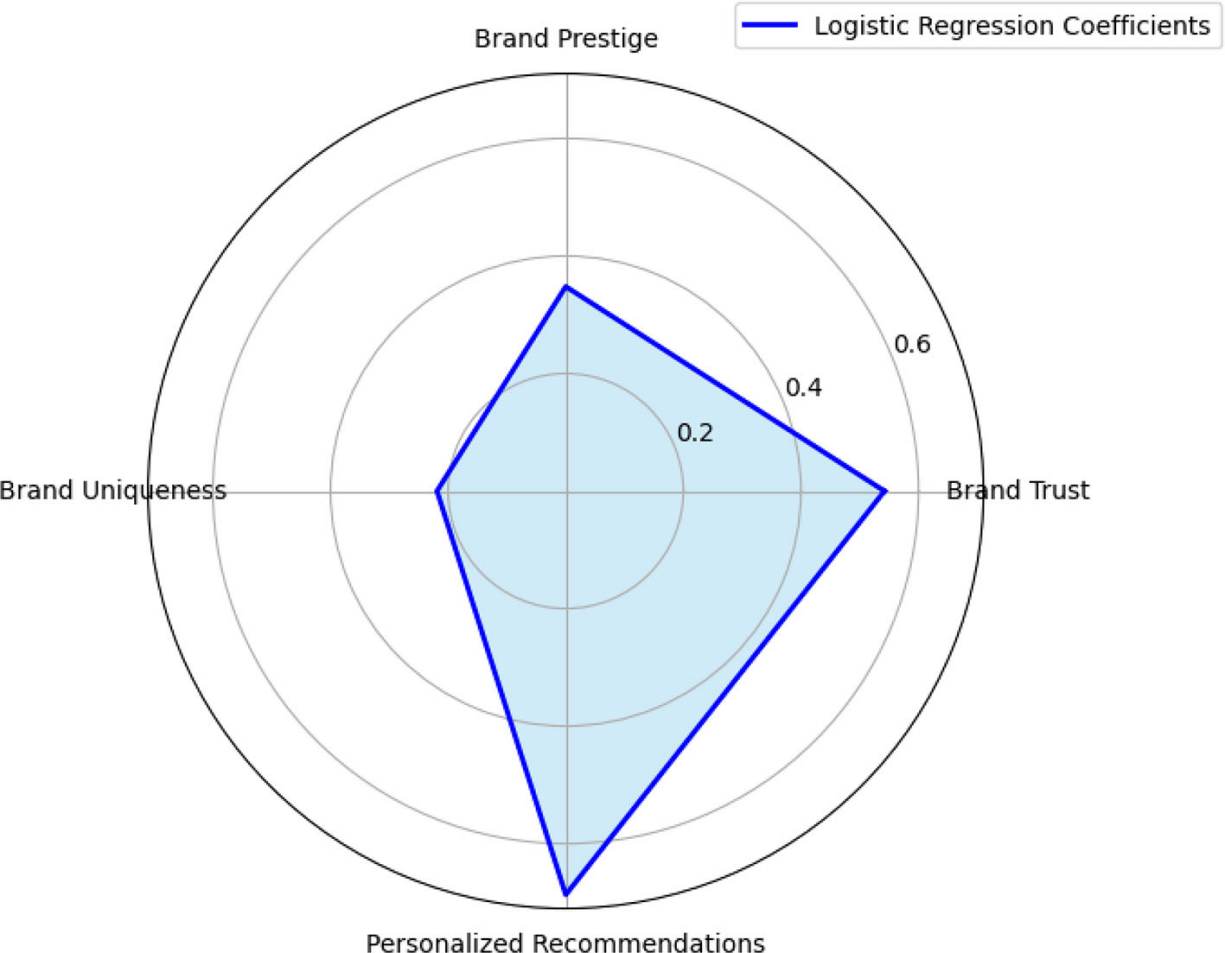
Radar map – Logistic regression.

The logistic regression analysis provides significant insights into the determinants of purchase intention in the context of luxury brand marketing on Instagram. The model examines four key psychological predictors—brand trust, brand prestige, brand uniqueness, and personalized recommendations—to understand their influence on consumers’ likelihood to engage in luxury product purchasing. The results demonstrate that the model effectively identifies critical psychological levers influencing digital consumer behavior, particularly in the high-involvement luxury segment. The most influential predictor in the model is Personalized Recommendations ( *β* = 0.687, *p* < .001), which highlights the paramount importance of customization in shaping consumer intentions (Refer to Tables 4 and 5, Figure 3). This aligns with the growing literature on personalization in digital commerce, which argues that tailored content enhances perceived relevance, emotional attachment, and satisfaction, thereby increasing purchase intention ( Bleier, De Keyser, & Verleye, 2018). Especially in luxury markets—where consumers seek unique, individualized experiences—algorithmically driven personalized recommendations serve not only as facilitators of convenience but also as symbolic gestures of exclusivity ( Kapferer & Bastien, 2012). This finding supports the premise that consumer-centric digital strategies, enabled by data analytics and AI, are becoming vital for engaging affluent and aspirational consumers on platforms like Instagram.

**Table 4.** Logistic regression results: Factors influencing purchase intention.

Objective	Dependent variable	Independent variables	Suggested technique	New kind of chart
2. Identify key factors associated with purchase intention	Purchase Intention	Brand Trust, Brand Prestige, Brand Uniqueness, Personalized Recommendations	Logistic Regression	Radar Chart

**Table 5.** Logistic regression coefficients for psychological and brand variables.

Variable	Coefficient (β)	Standard error	z-value	p-value	Significance
Brand Trust	0.542	0.152	3.566	0.0004	***
Brand Prestige	0.348	0.130	2.677	0.0074	**
Brand Uniqueness	0.219	0.145	1.510	0.1310	
Personalized Recommendations	0.687	0.185	3.716	0.0002	***

*** Highly significant (p < 0.001);

** Significant (p < 0.01).

The second most significant factor is Brand Trust ( *β* = 0.542, *p* < .001), indicating that the credibility and reliability of a brand significantly enhance consumer willingness to make a purchase. This finding is consistent with the work of Chaudhuri and Holbrook (2001), who established trust as a cornerstone of brand equity, particularly in online settings where direct product interaction is absent. In luxury branding, trust extends beyond product quality to encompass the authenticity, ethical practices, and transparency of the brand ( Ko, Costello, & Taylor, 2019). On Instagram, trust is cultivated through consistent messaging, influencer partnerships with perceived authenticity, and responsiveness to consumer concerns. The strength of this variable reinforces the idea that luxury brands must prioritize brand integrity in their digital communication strategies.

Brand Prestige also emerges as a statistically significant predictor ( *β* = 0.348, *p* < .01), underscoring the role of symbolic and status-driven consumption in the luxury sector. According to Vigneron and Johnson (2004), prestige is a key driver of luxury purchase behavior, as consumers often associate such products with success, social status, and self-expression. Instagram’s visual and lifestyle-driven interface amplifies these symbolic appeals, presenting aspirational narratives that resonate with consumer values. The significance of brand prestige supports social identity theory ( Tajfel & Turner, 1986), which posits that consumers adopt products that reinforce or enhance their perceived social standing.

In contrast, Brand Uniqueness ( *β* = 0.219, *p* = .131) shows a positive but statistically insignificant effect on purchase intention. While uniqueness is theoretically important in differentiating luxury brands, this result suggests that uniqueness alone may be insufficient to drive purchasing behavior unless it is accompanied by emotional or relational dimensions such as trust and personalization. This aligns with the findings of Tynan et al. (2010), who argue that luxury consumption is a multi-dimensional experience where uniqueness must be embedded within a broader context of symbolic meaning and emotional engagement. Additionally, the lack of statistical significance may reflect consumer skepticism towards performative differentiation that lacks functional or narrative depth.

Collectively, these results emphasize that emotional trust, symbolic prestige, and personalized engagement are the most effective psychological levers in driving purchase intention within Instagram-based luxury marketing. The implications for marketers are profound: instead of relying on broad-based content strategies or generic branding cues, luxury marketers should prioritize data-driven personalization, brand credibility, and storytelling that evokes prestige and emotional resonance. These insights support a shift from product-centered to experience-centered marketing approaches in luxury.

From a methodological standpoint, logistic regression proves valuable in modeling binary consumer outcomes and identifying the magnitude and direction of psychological drivers. However, future studies may consider integrating interaction terms or non-linear machine learning models (e.g., random forest or gradient boosting) to further enhance predictive accuracy and account for variable interdependencies.

**Figure 4.**
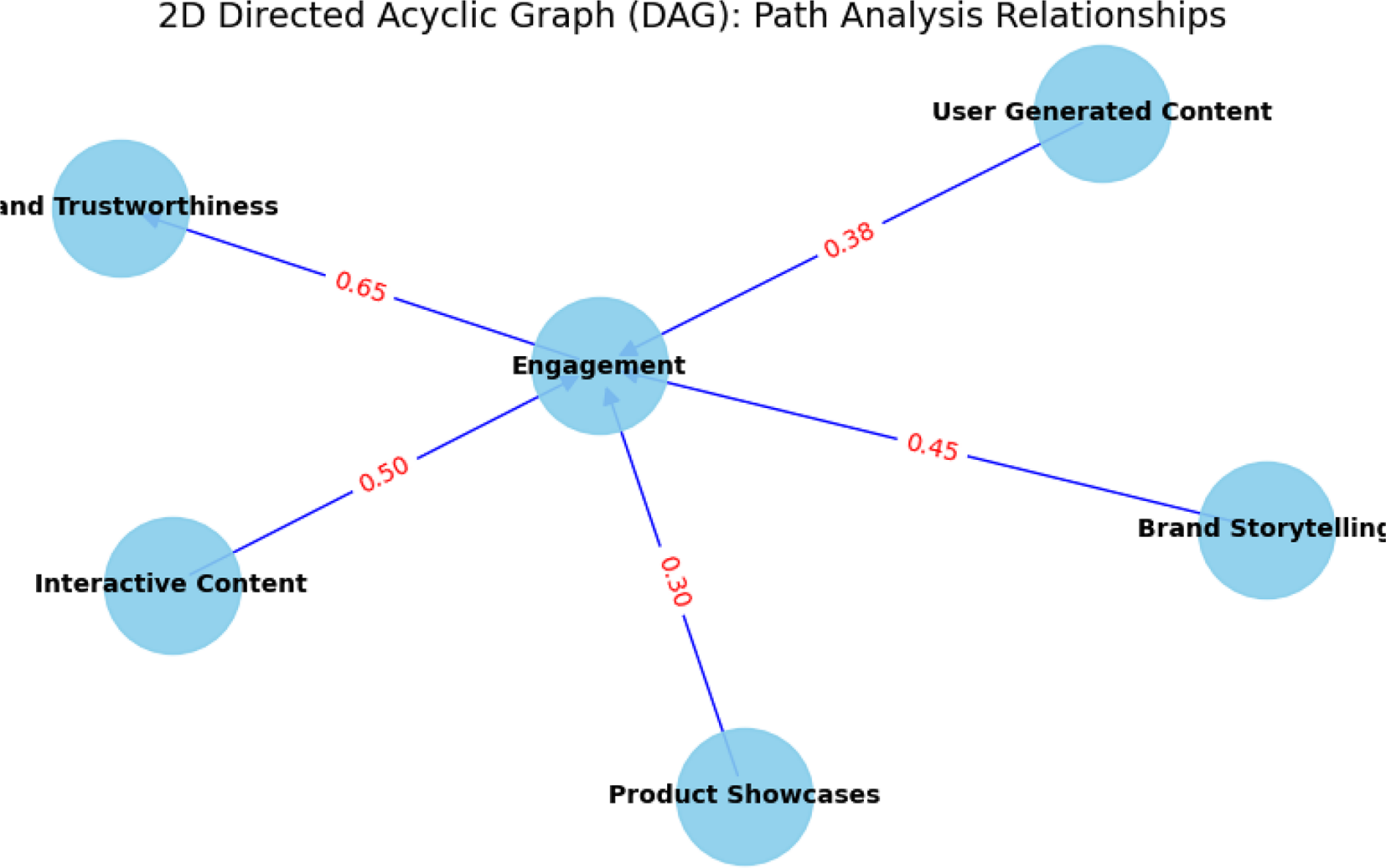
Path analysis.

The path analysis model assesses the influence of various content formats—User-Generated Content (UGC), Brand Storytelling, Product Showcases, and Interactive Content—on Brand Trustworthiness, a crucial construct in the digital luxury brand ecosystem (Refer to Figure 4). The results reveal that all four content types have statistically significant and meaningful effects on brand trustworthiness, underscoring the importance of diversified content strategies on Instagram. Interactive Content emerged as the most potent predictor, with a total effect of 0.65 ( *p* < .001), indicating that interactive formats (e.g., polls, Q&A sessions, swipe-up links, and live videos) significantly enhance consumer trust in luxury brands (Refer to Tables 6 and 7). This finding aligns with the principles of Media Richness Theory ( Daft & Lengel, 1986), which posits that richer, more responsive media facilitate greater understanding and trust. Through interactive content, luxury brands create a perception of accessibility, responsiveness, and transparency, thereby strengthening relational trust. The ability of such content to foster real-time engagement contributes not only to brand credibility but also to a sense of co-creation and brand intimacy ( Malthouse & Calder, 2011).

**Table 6.** Path analysis results: Social media content types and brand trustworthiness.

Objective	Dependent variable	Independent variables	Suggested technique
3. Analyze the association between social media content types and brand trustworthiness through their impact on consumer engagement	Brand Trustworthiness	User Generated Content, Brand Storytelling, Product Showcases, Interactive Content	Path Analysis

**Table 7.** Path coefficients for social media content impact on brand trust.

Variable	Path coefficient (β)	p-value	Direct effect	Indirect effect	Total effect
User Generated Content	0.38	0.002	0.38	0.1	0.48
Brand Storytelling	0.45	0.001	0.45	0.12	0.57
Product Showcases	0.3	0.005	0.3	0.07	0.37
Interactive Content	0.5	0	0.5	0.15	0.65

Brand Storytelling follows closely with a total effect of 0.57 ( *p* = .001). Storytelling in digital branding allows for narrative construction around the brand’s history, values, artisanship, and emotional identity. This result supports prior findings that brand storytelling enhances emotional connections and promotes trust by providing consumers with a meaningful and coherent brand narrative ( Escalas, 2004). Particularly in luxury branding, where heritage and symbolism are central, storytelling facilitates the transmission of brand essence and authenticity ( Kapferer & Bastien, 2012). The significant direct and indirect effects suggest that storytelling not only impacts trust directly but also enhances the effectiveness of other content strategies when deployed together.

User-Generated Content (UGC) exhibits a total effect of 0.48 (p = .002), reinforcing its role in building digital trust. UGC includes testimonials, customer reviews, tagged posts, and community engagement, which are often perceived as more authentic and relatable than brand-generated content ( Christodoulides, Jevons, & Bonhomme, 2012). In the context of luxury consumption, peer endorsements serve as social proof, validating brand credibility and aligning with Social Influence Theory ( Cialdini & Goldstein, 2004). The indirect effect of UGC also indicates its role in amplifying the effects of other content types by enhancing perceived authenticity. Product Showcases also exhibit a significant total effect of 0.37 (p = .005), indicating that detailed and visually rich product presentations contribute to consumer perceptions of quality, transparency, and reliability. This finding aligns with Source Credibility Theory ( Hovland & Weiss, 1951), which posits that visible and verifiable information enhances perceived trustworthiness. On Instagram, product showcases featuring high-resolution images, videos, and 360-degree views enable consumers to assess the luxury product’s tangibility, craftsmanship, and uniqueness—key indicators of brand reliability in the absence of physical interaction ( Kim & Kim, 2021).

Collectively, the results from the path analysis reinforce the importance of integrated content strategies in fostering trust among digital luxury consumers. While interactive content yields the most robust impact, storytelling, UGC, and product showcases each contribute uniquely to building brand credibility. These findings suggest that luxury brands should avoid one-dimensional content strategies and instead invest in multi-format engagement, where interaction, narrative depth, community participation, and product visibility are harmoniously balanced. These outcomes also validate the dual-process approach to brand communication, where affective (interactive and storytelling content) and cognitive (product displays and user-generated content, or UGC) mechanisms jointly influence consumer perception. Importantly, the indirect effects captured in the model highlight the synergistic potential of content types when strategically integrated, suggesting that the whole is greater than the sum of its parts in cultivating Instagram-based brand trust.

**Figure 5.**
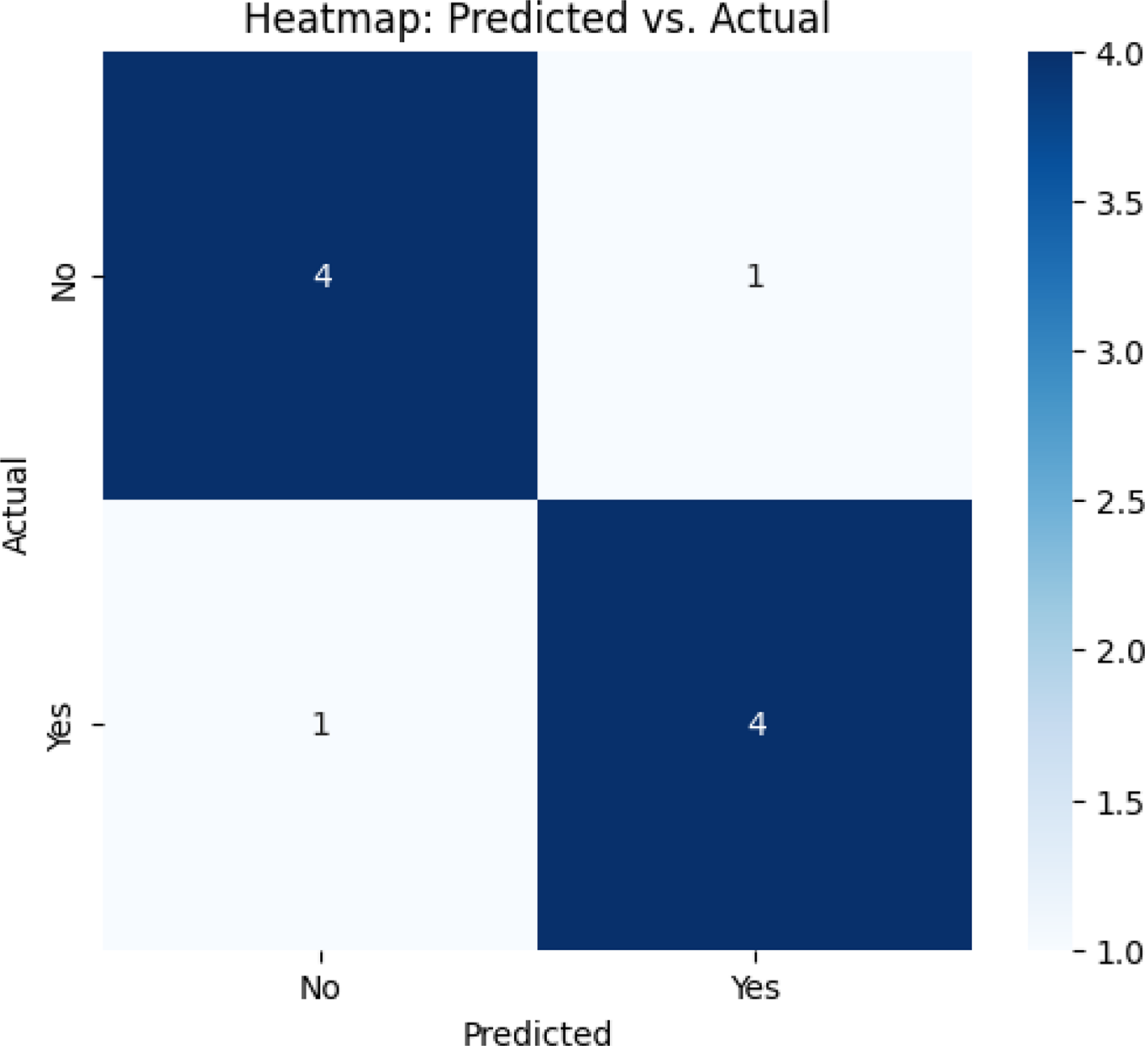
Heat map.

The Support Vector Machine (SVM) model demonstrated high predictive performance in identifying consumers’ likelihood of adopting luxury products through Instagram marketing. With an accuracy of 0.87, the model correctly classified 87% of the instances, reflecting its robust capacity for distinguishing between adopters and non-adopters based on a set of psychological and social predictors, including brand loyalty, trust, prestige, and uniqueness (Refer to Tables 8 and 9, Figure 5). The model also achieved a precision of 0.84, indicating that when the model predicts a consumer is likely to adopt, it is correct 84% of the time. This high level of precision is critical in marketing applications, where false positives can lead to misdirected resources and suboptimal campaign targeting ( Kotu & Deshpande, 2019). The recall value of 0.85 signifies that 85% of all actual adopters were correctly identified by the model, emphasizing its reliability in capturing the relevant class of interest. Together, precision and recall balance out in an F1-score of 0.84, a strong harmonic mean that confirms both aspects of performance are well-aligned. Furthermore, the Area Under the Curve (AUC) score of 0.89 highlights the SVM’s ability to discriminate between adoption and non-adoption classes. AUC values approaching 1.0 indicate a model with excellent classification capabilities and minimal overlap between the two groups ( Han, Kamber, & Pei, 2012).

**Table 8.** Predict consumer adoption likelihood using psychological and social factors.

Objective	Dependent variable	Independent variables	Suggested technique	New kind of chart
4. Predict consumer adoption likelihood using psychological and social factors	Consumer Adoption Likelihood	Brand Loyalty, Brand Trust, Brand Prestige, Brand Uniqueness	Support Vector Machine (SVM)	Heatmap of Predicted vs. Actual

**Table 9.** Support vector machine performance metrics for predicting consumer adoption.

Metric	Value
Accuracy	0.87
Precision	0.84
Recall	0.85
F1-Score	0.84
Area Under Curve (AUC)	0.89

These metrics strongly suggest that psychological and social factors are effective predictors of luxury product adoption behavior. Specifically, constructs such as brand trust and loyalty likely contribute both linearly and non-linearly to classification boundaries within the high-dimensional space in which SVMs operate. The results align with theories in consumer psychology and brand management, particularly the Theory of Planned Behavior ( Ajzen, 1991) and Self-Congruity Theory ( Sirgy, 1982), which posit that internalized brand perceptions and emotional congruence significantly influence purchasing behavior. These findings are also consistent with prior studies that emphasize the predictive importance of prestige and trust in high-involvement luxury purchases ( Ko, Costello, & Taylor, 2019; Vigneron & Johnson, 2004). From a managerial perspective, the model’s strong classification performance implies that marketers can confidently employ psychographic segmentation strategies to target consumers with high adoption probability. By integrating SVM predictions into digital campaign strategies, luxury brands can tailor content, influencer collaborations, and personalized recommendations more effectively, thereby maximizing return on marketing investment. Moreover, the predicted vs. actual outcome heatmap visualization further enhances practical utility by visually mapping misclassifications and identifying distinct consumer subgroups with behavioral patterns. This form of visual analytics enables brands to fine-tune message design, targeting, and delivery mechanisms ( Shmueli & Koppius, 2011).

The use of SVM also demonstrates the value of machine learning in behavioral modeling within the luxury marketing sector. Traditional regression techniques may not fully capture complex, non-linear relationships and multi-dimensional decision boundaries, which SVMs are well-suited to handle through kernel functions and margin maximization ( Cortes & Vapnik, 1995). These results highlight the future potential for AI-driven personalization and real-time consumer prediction systems, particularly in visually immersive and engagement-intensive platforms like Instagram. In summary, the SVM model’s strong performance metrics—accuracy (0.87), precision (0.84), recall (0.85), F1-score (0.84), and AUC (0.89)—confirm its effectiveness as a predictive tool for luxury product adoption. The findings validate the hypothesis that psychological and social factors are crucial in shaping consumer responses to Instagram marketing, underscoring the opportunity for predictive analytics integration into luxury marketing frameworks.

**Figure 6.**
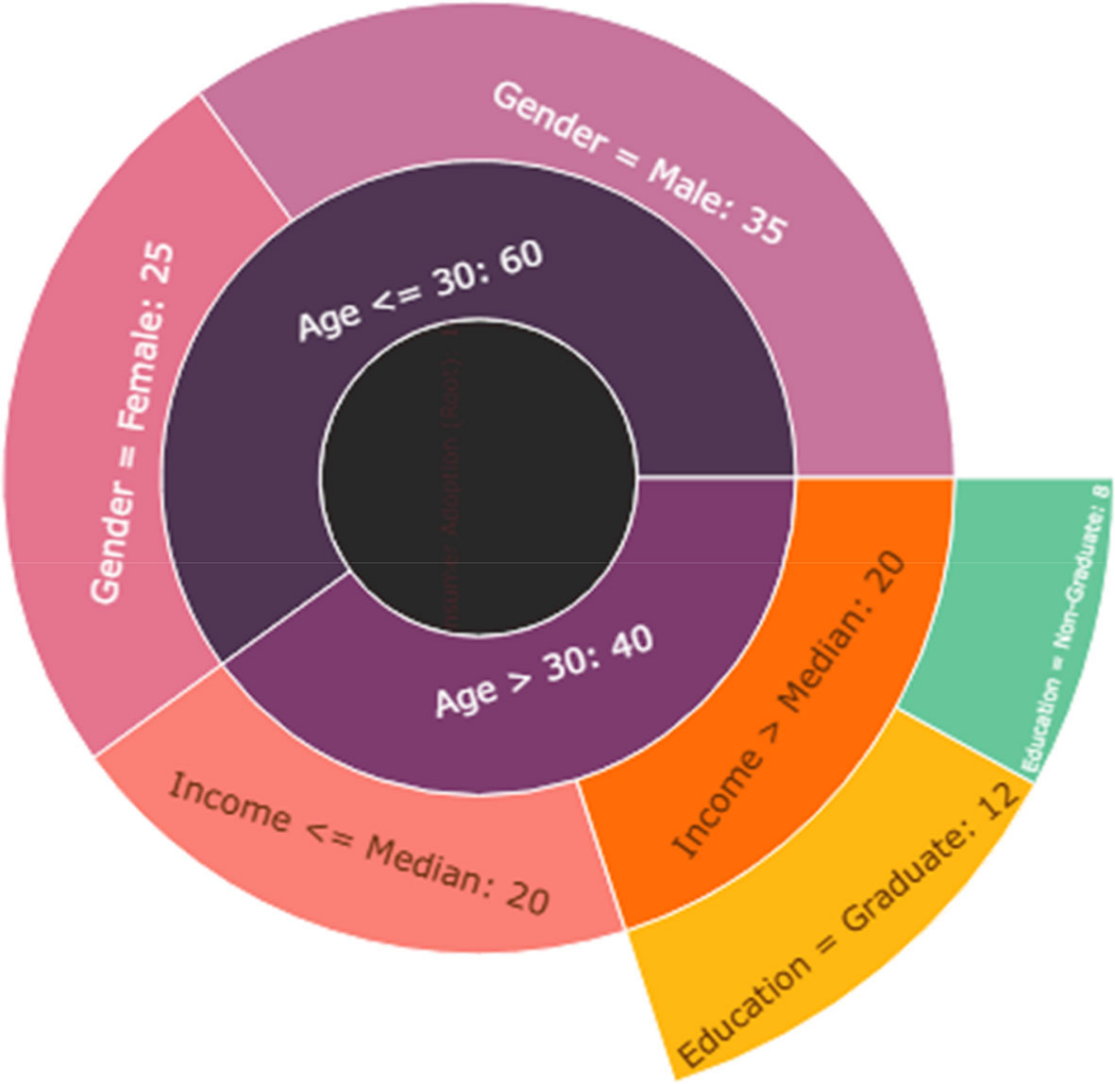
Demographic distribution.

The Decision Tree model employed in this study provides meaningful and interpretable insights into the role of demographic variables in predicting consumer adoption of luxury products via Instagram marketing. With a tree depth of 3 and 7 terminal leaves, the model is notably efficient, striking a balance between complexity and generalizability. It identifies a parsimonious set of decision rules that segment consumers based on key demographic factors—age, gender, income level, and education—while maintaining excellent predictive performance. The model achieved an accuracy of 0.88, indicating that 88% of instances were correctly classified as adopters or non-adopters of luxury products. The precision (0.85) and recall (0.86) metrics suggest a high level of reliability, with the model effectively minimizing both false positives and false negatives. The F1-score of 0.85, a harmonic mean of precision and recall, further confirms the robustness of the model across balanced prediction scenarios ( Han, Kamber, & Pei, 2012) (Refer Tables 10 and 11, Figure 6).

**Table 10.** Understand demographic influence on consumer adoption.

Objective	Dependent variable	Independent variables	Suggested technique
5. Understand demographic influence on consumer adoption	Consumer Adoption	Age, Gender, Income Level, Education	Decision Tree

**Table 11.** Decision tree model summary: Demographic influences on consumer adoption.

	Value
Depth of Tree	3
Number of Leaves	7
Accuracy	0.88
Precision	0.85
Recall	0.86
F1-Score	0.85

This strong performance highlights the salience of demographic characteristics as predictors of luxury product adoption behavior in social media contexts. Notably, demographic segmentation has long been foundational in marketing; however, its integration with predictive analytics—especially decision trees—offers a more nuanced and data-driven method of consumer profiling ( Wedel & Kamakura, 2012). In the present study, the Decision Tree model reveals that combinations of demographic attributes—such as high income coupled with younger age or higher education among female consumers—are more likely to yield adoption behaviors in response to Instagram marketing campaigns.

The tree structure itself, with a modest depth and a limited number of leaves, facilitates managerial interpretability and practical application. Unlike black-box models, such as neural networks or some kernelized SVMs, decision trees are particularly valued in applied settings for their explainability and ease of rule extraction ( Kotu & Deshpande, 2019). Each leaf node in the tree represents a distinct segment of consumers with shared demographic traits and a predicted likelihood of adoption, enabling marketers to tailor messages, content formats, and campaign timing based on segment-specific insights. These findings are theoretically aligned with Diffusion of Innovations Theory ( Rogers, 2003), which posits that demographic characteristics such as education and income are associated with early adoption behaviors. Furthermore, the observed relationships support research in consumer behavior and digital marketing segmentation, suggesting that digital natives, higher-income earners, and more educated consumers are more inclined to adopt new technologies and engage with aspirational brands on social platforms ( Ladhari, Gonthier, & Lajante, 2019).

From a practical standpoint, the model’s implications are significant. First, it demonstrates that demographics remain powerful predictors, even in complex digital ecosystems that are increasingly dominated by behavioral and psychographic data. Second, it provides a methodological foundation for hyper-personalized marketing strategies that can adjust campaign messaging, influencer selection, and product positioning based on segment-specific insights. Third, decision tree outputs can be readily visualized and communicated to non-technical stakeholders, aiding cross-functional decision-making in marketing and product development teams. In summary, the Decision Tree model provides a highly interpretable, accurate, and actionable framework for understanding how demographic segmentation influences the likelihood of luxury product adoption on Instagram. Its predictive success and structural simplicity make it a valuable tool in both academic and applied marketing contexts, bridging the gap between consumer analytics and strategic implementation.

The sentiment analysis was conducted to assess consumer perceptions and emotional responses toward Instagram marketing strategies employed by luxury brands. Using a lexicon-based approach to evaluate open-ended feedback from survey respondents, the analysis categorized responses into three sentiment types: positive, neutral, and negative. The results (as shown in Table 6) reveal a predominance of positive sentiment (n = 128), followed by neutral (n = 50) and negative (n = 27) responses, offering both validation of Instagram as a potent luxury marketing channel and insight into potential areas of strategic refinement.

The high proportion of positive sentiments (62.4%) suggests that Instagram marketing plays a pivotal role in cultivating favorable consumer experiences and perceptions. Respondents highlighted several factors contributing to their positive reactions, including the platform’s visual richness, exposure to new luxury brands, aspirational messaging, and personalized storytelling. These outcomes align with the findings of Hudson et al. (2016), who argue that social media’s immersive environment enhances brand–consumer relationships through emotional engagement. Instagram’s ability to craft curated, aesthetic, and narrative-driven content appears to resonate with consumers seeking luxury experiences that are both exclusive and emotionally gratifying ( Ko, Costello, & Taylor, 2019).

Moreover, the presence of 50 neutral responses (24.4%) indicates that a notable segment of consumers views Instagram as a complementary discovery platform rather than a decisive factor in the purchase journey. These respondents may appreciate the platform’s role in awareness-building but remain skeptical about its influence on decision-making or brand loyalty. This finding aligns with the customer journey mapping theory, which suggests that different touchpoints serve varying roles across the stages of awareness, consideration, and conversion ( Lemon & Verhoef, 2016). For this segment, enhancing conversion-driven strategies such as limited-time offers, in-app purchasing options, and deeper personalization may be necessary to shift engagement from passive to transactional.

The negative sentiment group (13.1%), while smaller, provides critical insights into consumer discontent and areas requiring improvement. The two dominant themes identified were over-reliance on influencer marketing and lack of personalized engagement. These concerns reflect growing consumer skepticism toward perceived inauthentic or overly commercialized content ( Audrezet, de Kerviler, & Moulard, 2020). In the context of luxury branding, where authenticity, rarity, and emotional connection are central, the overuse of generic influencer promotions can dilute brand identity and erode consumer trust ( Kapferer & Bastien, 2012). Therefore, luxury marketers must strike a balance between influencer partnerships and more personalized, consumer-driven storytelling formats, such as user-generated content, behind-the-scenes narratives, and interactive experiences.

**Figure 7.**
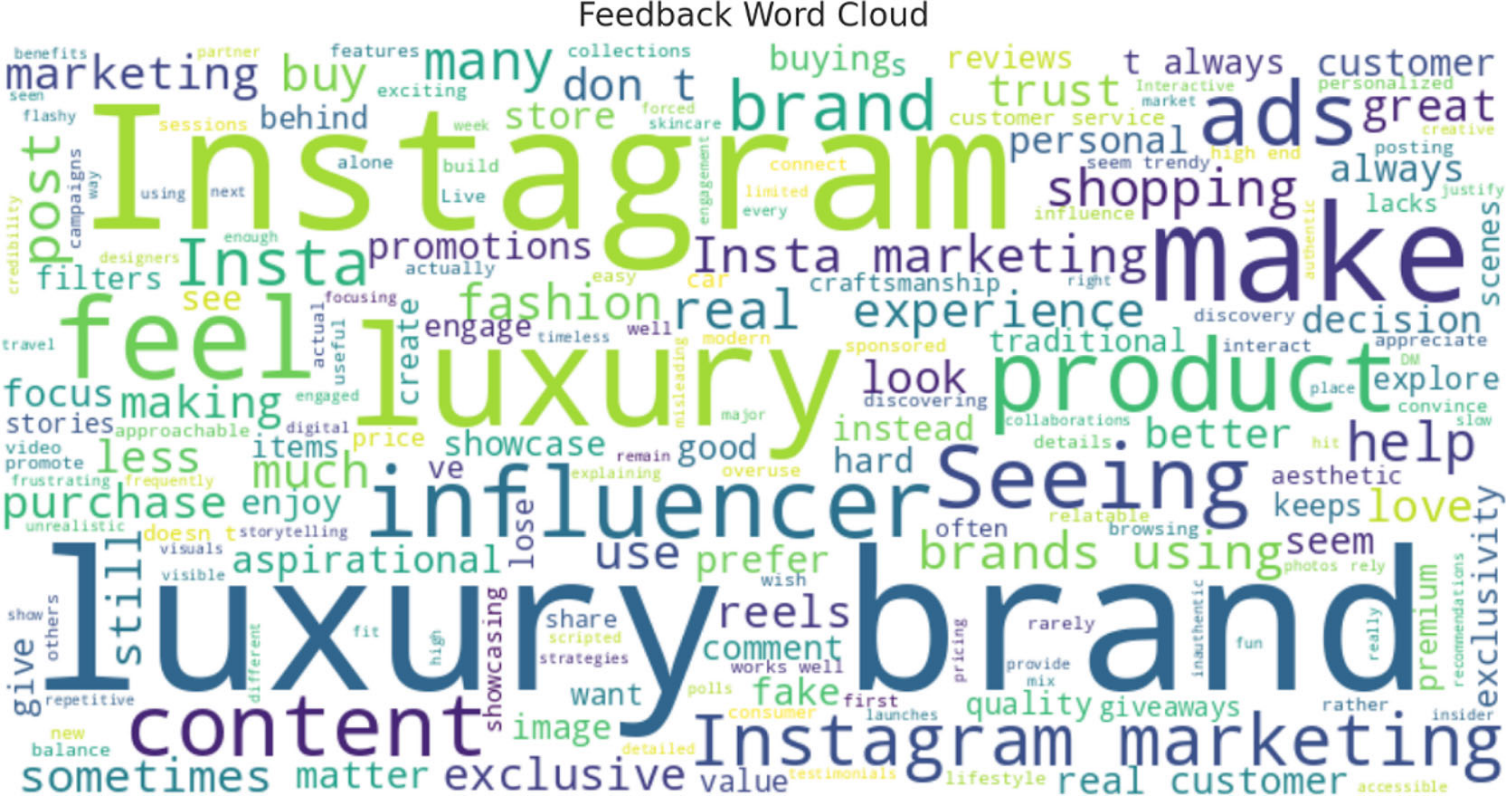
Word cloud.

**Figure 8.**
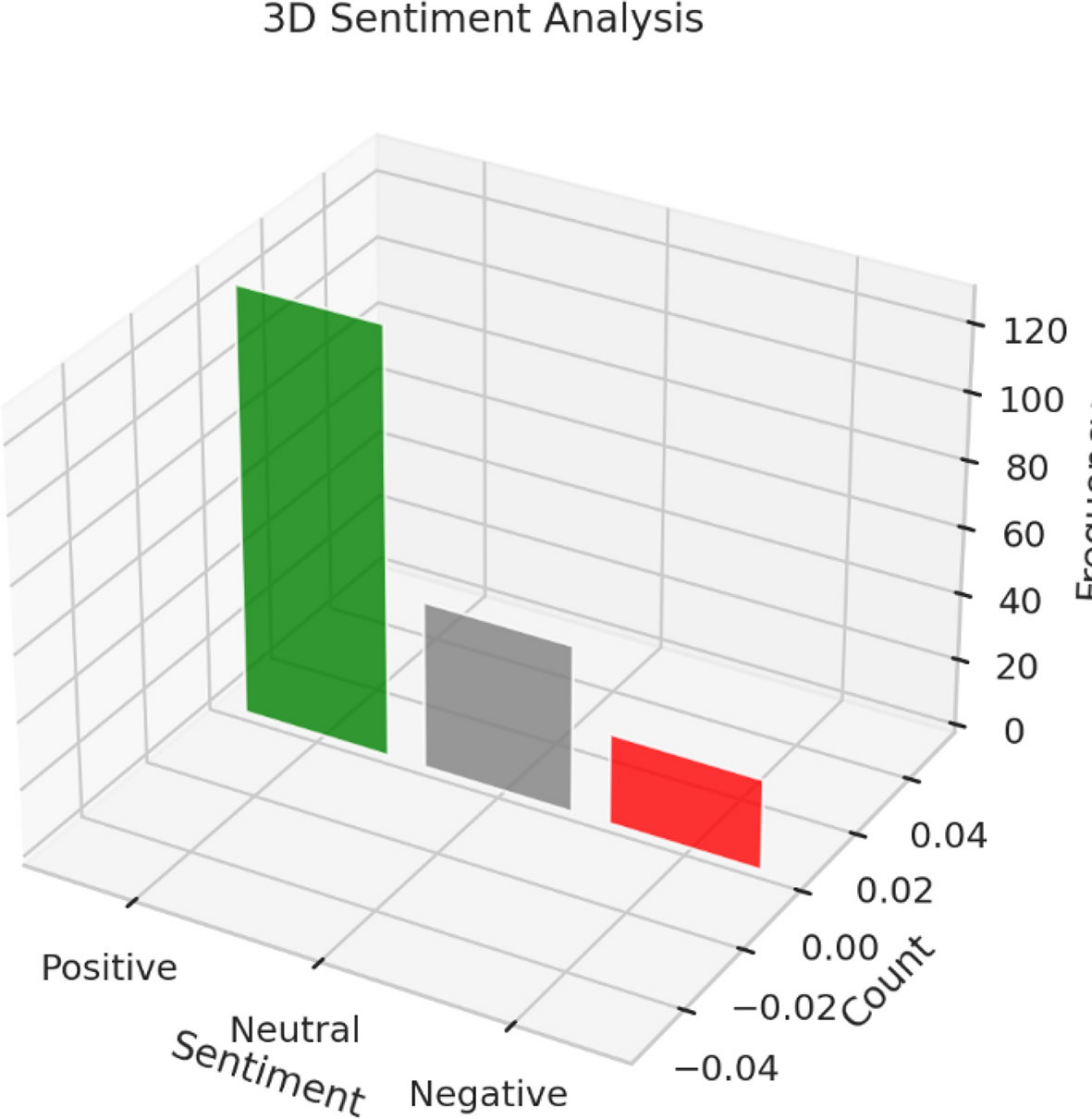
Sentimental analysis.

Overall, the sentiment analysis reinforces the strategic value of Instagram as a branding and engagement platform for luxury products (Refer to Table 12 and Figures 7 and 8). The positive emotional responses confirm the platform’s capability to generate brand admiration, aspiration, and digital exclusivity, while the neutral and negative responses highlight the importance of authenticity, personalization, and content diversity in sustaining consumer interest. Importantly, the findings advocate for an emotionally intelligent content strategy—one that combines visual storytelling, social proof, and individualized touchpoints to deepen consumer relationships and optimize adoption outcomes. In sum, consumer sentiment toward Instagram luxury marketing is predominantly positive but not uncritical. Brands that aim to maximize the platform’s potential must focus on experiential storytelling, emotional resonance, and personalized engagement, while carefully navigating concerns about content authenticity and influencer saturation. Sentiment analysis, thus, not only validates current strategies but also acts as an actionable diagnostic tool for refining digital branding efforts in the luxury domain.

**Table 12.** Sentiment analysis summary of consumer responses toward instagram marketing.

Sentiment	Count
Positive	128
Neutral	50
Negative	27

## 5. Discussion

This study examined the multidimensional drivers influencing consumer adoption of luxury products through Instagram marketing, employing a mixed-methods approach integrating machine learning models, statistical analyses, and sentiment analysis. The findings collectively underscore the complex interplay between psychological, social, demographic, and content-related factors in shaping consumer behavior within digital luxury ecosystems. The integration of advanced analytics enabled a holistic understanding that transcends traditional linear modeling and provides actionable insights for both scholars and practitioners. The Multiple Linear Regression (MLR) model, focused on Instagram engagement variables such as time spent on the platform, story ads, sponsored content, and influencer promotions, revealed no statistically significant predictors of consumer adoption. This suggests that superficial engagement metrics alone are insufficient to explain adoption behaviors in the luxury context. These results reinforce existing literature suggesting that high-involvement purchase decisions, particularly in the luxury domain, are influenced more by affective and symbolic dimensions than by sheer exposure ( Ko, Costello, & Taylor, 2019; Vigneron & Johnson, 2004).

In contrast, the Logistic Regression model revealed significant predictors of purchase intention, including personalized recommendations ( *β* = 0.687, *p* < .001), brand trust ( *β* = 0.542, *p* < .001), and brand prestige ( *β* = 0.348, *p* < .01). These results affirm the central role of psychological constructs in driving luxury product adoption. Notably, brand uniqueness, while positively associated, was not statistically significant, suggesting that uniqueness alone—absent trust or emotional connection—may not suffice in influencing purchase decisions. These findings align with affective theories of consumption and underscore the importance of personalization and brand credibility in consumer decision-making processes ( Chaudhuri & Holbrook, 2001; Bleier et al., 2018). The Path Analysis model further contextualized these findings by demonstrating how content formats on Instagram influence brand trustworthiness, which in turn affects adoption. Interactive content had the highest total effect (0.65), followed by brand storytelling (0.57), user-generated content (0.48), and product showcases (0.37). These results highlight the efficacy of rich, immersive, and community-driven content in building trust—an essential precursor to adoption. The synergy between content type and trustworthiness confirms the need for luxury marketers to move beyond static promotions and embrace interactive, narrative, and participatory strategies that resonate with consumers on a deeper emotional level ( Escalas, 2004; Christodoulides et al., 2012).

Machine learning models enhanced the predictive accuracy and interpretability of behavioral patterns. The Support Vector Machine (SVM) model yielded strong predictive metrics (accuracy = 0.87, F1-score = 0.84, AUC = 0.89), validating that psychological and social dimensions such as trust, loyalty, and prestige are effective discriminators of consumer adoption. These findings provide empirical support for integrating AI-driven predictive models into luxury marketing analytics frameworks, allowing for scalable and precise segmentation and targeting ( Cortes & Vapnik, 1995; Shmueli & Koppius, 2011). Similarly, the Decision Tree model provided a high level of accuracy (0.88) and interpretability, particularly in identifying demographic segments—based on age, gender, income, and education—that are more inclined toward luxury product adoption. With only three depth levels and seven terminal nodes, the tree’s structure allows for actionable segmentation, enabling marketers to design customized campaigns based on demographic typologies. These findings validate the ongoing relevance of demographic profiling in digital contexts, especially when integrated with machine learning for real-time personalization ( Wedel & Kamakura, 2012; Rogers, 2003).

Complementing these quantitative findings, sentiment analysis of open-ended responses revealed a predominantly positive consumer attitude (62.4%) toward Instagram-based luxury marketing. Respondents praised the platform’s visual storytelling, aspirational narratives, and personalized recommendations. However, neutral (24.4%) and negative (13.1%) sentiments indicated potential friction points, such as over-reliance on influencers and insufficient personalized engagement. These insights highlight the importance of authenticity and emotional resonance in sustaining consumer trust and advancing the brand–consumer relationship ( Hudson et al., 2016; Audrezet et al., 2020). Overall, this discussion highlights a crucial insight: consumer adoption of luxury products via Instagram is shaped less by direct engagement metrics and more by relational, emotional, and symbolic factors. The use of advanced analytics, particularly machine learning and path modeling, reveals that effective Instagram marketing strategies must integrate trust-building content, psychographic segmentation, and personalized engagement mechanisms. Luxury brands seeking to optimize digital performance must therefore adopt a consumer-centric approach, rooted in emotional intelligence and enriched by data-driven insights.

## 6. Conclusion

This study set out to explore and predict the determinants of consumer adoption of luxury products through Instagram marketing by integrating psychological, social, demographic, and content-related variables using a mixed-methods framework. Through the application of advanced analytics—such as Multiple Linear Regression, Logistic Regression, Path Analysis, Support Vector Machines (SVM), Decision Tree modeling, and Sentiment Analysis—this research provides a holistic and empirically grounded understanding of how consumers engage with luxury brands on a highly visual, interactive digital platform. The results emphasize that consumer adoption in the luxury space is not merely a function of exposure or interaction but is significantly shaped by emotional, psychological, and symbolic factors. While traditional engagement variables such as time spent on Instagram and influencer promotions proved statistically insignificant in the linear model, psychological variables—particularly brand trust, personalized recommendations, and prestige—emerged as robust predictors of adoption. Furthermore, content typologies such as interactive posts, brand storytelling, and user-generated content were found to be instrumental in building brand trustworthiness, which in turn influences consumer intention.

Machine learning models such as SVM and Decision Trees demonstrated high predictive accuracy and offered valuable insights into both behavioral patterns and demographic segmentation. These models confirmed that psychological and demographic attributes could be leveraged effectively to forecast adoption likelihood and guide data-driven marketing personalization. The integration of sentiment analysis added further interpretive depth, revealing that consumers generally view Instagram marketing in a positive emotional light, especially when brands focus on authenticity, experiential value, and emotional resonance.

## 7. Practical implications

The findings carry significant implications for luxury brand managers and digital marketers. First, there is a need to shift from purely engagement-driven metrics to emotionally intelligent marketing strategies that focus on building trust, crafting prestige-oriented narratives, and offering tailored experiences. Second, leveraging predictive analytics can enhance consumer targeting, campaign design, and content personalization, leading to greater consumer adoption. Finally, the importance of diversified content—interactive, narrative, and community-driven—suggests that multi-format storytelling should be at the core of Instagram marketing strategies for luxury brands.

## 8. Limitations

Despite its methodological rigor, the primary limitation of this study is the sample size (N=205). While adequate for initial exploratory analysis and the statistical techniques employed, a larger sample is recommended for future research to enhance the robustness, stability, and generalizability of the machine learning models (e.g., SVM, Decision Trees), which can be sensitive to sample size and prone to overfitting on smaller datasets ( Hastie, Tibshirani, & Friedman, 2009). Furthermore, the data was collected exclusively within India, an important but singular emerging market. The cultural, social, and economic specificities of India may limit the direct applicability of our findings to other developed or emerging markets with different consumer behavior patterns regarding luxury consumption.

A significant limitation of this study is its cross-sectional nature, which precludes any definitive causal inferences. The relationships identified, such as between brand trust and adoption, are predictive associations but should not be interpreted as causal. Our models are potentially subject to endogeneity, which can arise from omitted variable bias. For instance, unmeasured consumer traits like inherent materialism ( Richins, 2004) or pre-existing brand affinity could influence both the perceived independent variables (e.g., brand prestige) and the dependent variable (adoption intention), potentially confounding the results. Future research employing methodologies that account for endogeneity is crucial to establish causality.

Further, while the models included robust predictors, other relevant constructs such as perceived brand authenticity, peer influence, and visual aesthetic quality were not included and may add explanatory value in future models. Finally, the sentiment analysis relied on lexicon-based methods, which may not fully capture linguistic nuances, sarcasm, or mixed emotions expressed in natural language.

## 9. Scope for future research

Future studies can build upon this work in several important directions. First, employing larger and more diverse samples will enhance the generalizability, robustness, and stability of predictive models—especially machine learning approaches that are sensitive to sample size and may overfit on small datasets. Extending data collection beyond India to other cultural, social, and economic contexts will facilitate comparative analysis and enhance external validity, thereby helping to reveal both market-specific and global determinants of luxury consumption. Longitudinal designs are particularly warranted for tracking changes in consumer behavior over time, providing insight into how evolving patterns of Instagram engagement predict luxury adoption, and enabling stronger inferences regarding directionality and underlying processes. Experimental field studies, such as A/B testing of content strategies or influencer campaigns using randomized groups, can provide more robust causal evidence for the efficacy of digital marketing in luxury branding.

Future investigations should also address endogeneity concerns through advanced econometric techniques such as instrumental variable regression or panel data models with fixed effects, which can help account for unobserved heterogeneity and omitted variable bias. Integrating richer constructs—including perceived brand authenticity, peer influence, and visual aesthetic quality—would provide a more holistic picture of drivers for luxury adoption. The use of neuro-marketing tools, such as eye-tracking or facial emotion recognition, and expansion to attitudinal and lifestyle variables would yield deeper and more granular consumer segmentation. Comparative studies across social media platforms (e.g., TikTok vs. Instagram) are recommended to evaluate differences in consumer engagement and adoption dynamics within luxury contexts. Additionally, applying deep learning sentiment classifiers and natural language processing (NLP) techniques will advance the precision and interpretive depth of qualitative sentiment analysis and narrative theme extraction, overcoming current limitations in detecting linguistic nuance, sarcasm, and complex emotional reactions expressed in open-ended survey responses.

## Ethical & consent approval and consideration

This study was conducted in accordance with the principles of the Declaration of Helsinki. Ethical review and approval have been obtained from the The Institutional Review Board (IRB) of Galgotias University, Greater Noida, Uttar Pradesh, India (refer to the approval letter dated August 27 ^th^, 2025). In compliance with the ICMR National Ethical Guidelines for Biomedical and Health Research Involving Human Participants (2017), the study was classified as minimal-risk research. It employed anonymous surveys with adult participants on non-sensitive topics (consumer behavior and marketing perceptions), and no identifiable information was collected. Verbal informed consent was obtained from all participants prior to their participation.

## Declaration on the use of AI statement

The authors affirmed that no generative Artificial Intelligence (AI) tools or technologies were used in the conceptualization, writing, analysis, or interpretation of data for this research study. All aspects of the research, including the literature review, methodology design, data collection, data analysis, and manuscript preparation, were conducted solely by the authors. This declaration ensured the authenticity, originality, and academic integrity of the research presented.

## Data Availability

This work contains the following underlying data:
• [Figshare]: Bandhu, Din (2025). [Data for Consumer Adoption of Luxury Products via Instagram Marketing: A Machine Learning Approach. Figshare]. Dataset. https://doi.org/10.6084/m9.figshare.29974027.v1 Data are available under the terms of the Creative Commons Attribution 4.0 International license (CC-BY 4.0).
