# Supplementary material for: Predicting Consumer Adoption of Luxury Products via Instagram Marketing: A Machine Learning Approach
Source: F1000Res. 2025 Oct 23;14:890. Originally published 2025 Sep 9. [Version 2] doi: 10.12688/f1000research.169639.2 (PMC12603530; doi:10.12688/f1000research.169639.2)
Supplement: Peer review history

# Reviewer report: Reviewer response for version 2

**DOI:** 10.5256/f1000research.189452.r427387

**Published:** 2025-11-19

**Authors:** Ramachandran K K

**Affiliations:** 1 GRD Institute of Management, Coimbatore, India

This paper investigates how Instagram marketing influences the adoption of luxury products, emphasizing the intersection of technological innovation, consumer psychology, and digital engagement. By employing both machine learning models and traditional statistical analyses, the study effectively highlights the predictive relationships among Instagram engagement metrics, brand trust, prestige, and personalized recommendations in shaping consumer purchase behaviour. It reveals that, while surface-level engagement provides limited predictive value, psychological and brand-related factors play a crucial role in determining consumer intention and adoption.

 The research successfully integrates theories such as the Theory of Planned Behaviour, Elaboration Likelihood Model, and Social Identity Theory to provide a strong conceptual foundation. It also utilizes a mixed-methods approach—combining quantitative analysis with sentiment evaluation—to offer a comprehensive view of digital consumer behaviour. However, the study’s relatively small sample size and its exclusive focus on Instagram limit the broader generalizability of its findings.

 Despite these constraints, the paper contributes valuable insights into how emotional resonance, influencer authenticity, and demographic factors affect luxury brand adoption. Its methodological rigor, practical implications, and interdisciplinary approach make it a timely and significant contribution to the fields of digital marketing and consumer behavior, offering strategic guidance for brands aiming to optimize their social media marketing efforts and enhance consumer engagement in the luxury segment.

Is the work clearly and accurately presented and does it cite the current literature?

Yes

If applicable, is the statistical analysis and its interpretation appropriate?

Yes

Are all the source data underlying the results available to ensure full reproducibility?

Yes

Is the study design appropriate and is the work technically sound?

Yes

Are the conclusions drawn adequately supported by the results?

Yes

Are sufficient details of methods and analysis provided to allow replication by others?

Yes

Reviewer Expertise:

Marketing Management, Brand Management, Strategic Marketing, Promotion, Management, Professional Career Management, Principles of Management, Retail Marketing, Services Marketing, Social Media Marketing, International Marketing, Event Marketing, Sports marketing and Consumer Behavior, Principles of Accounting, Financial Accounting, Management Accounting, Cost Accounting, Artificial Intelligence, Big Data Processing in Digital Marketing Platforms

I confirm that I have read this submission and believe that I have an appropriate level of expertise to confirm that it is of an acceptable scientific standard.

## References

[1] : 12Enhancing Business Development, Ethics, and Governance with the Adoption of Distributed Systems.2024; 10.1002/9781394188093.ch12 193-209 10.1002/9781394188093.ch12

# Reviewer report: Reviewer response for version 2

**DOI:** 10.5256/f1000research.189452.r426396

**Published:** 2025-11-10

**Authors:** Singgih Santoso

**Affiliations:** 1 Universitas Kristen Duta Wacana Yogyakarta, Yogyakarta, Indonesia

The revisions and responses provided by the authors have generally addressed the previous comments and questions satisfactorily. However, it is suggested that in the *Discussion* section, the authors explicitly state which hypotheses (H1–H8) are supported or not supported, so that readers can clearly follow the logical flow between the hypothesis development and the discussion.

Is the work clearly and accurately presented and does it cite the current literature?

Yes

If applicable, is the statistical analysis and its interpretation appropriate?

Partly

Are all the source data underlying the results available to ensure full reproducibility?

Yes

Is the study design appropriate and is the work technically sound?

Yes

Are the conclusions drawn adequately supported by the results?

Yes

Are sufficient details of methods and analysis provided to allow replication by others?

Yes

Reviewer Expertise:

consumer behavior, digital marketing

I confirm that I have read this submission and believe that I have an appropriate level of expertise to confirm that it is of an acceptable scientific standard.

# Reviewer report: Reviewer response for version 1

**DOI:** 10.5256/f1000research.186997.r413333

**Published:** 2025-09-30

**Authors:** Dilip D

**Affiliations:** 1 VIT Business School, Vellore, Tamil Nadu, India

This study explores the impact of Instagram on luxury brand adoption, analyzing data from 205 respondents to identify key drivers of purchase intention and brand trustworthiness. The findings reveal that psychological and social variables, such as brand trust, prestige, uniqueness, and personalized recommendations, strongly influence consumer behavior, while surface-level engagement metrics have limited predictive power. A Support Vector Machine (SVM) model accurately predicts adoption likelihood with 87% accuracy, and sentiment analysis shows predominantly positive consumer attitudes toward Instagram marketing strategies. The study offers actionable insights for marketers, highlighting the importance of authenticity, emotional connection, and targeted psychographic segmentation in luxury digital marketing.

 Questions :

1. What are the implications of using a sample size of 205 for Structural Equation Modeling (SEM) analysis, and how might this impact the reliability of the findings?
2. How does the sample size of 205 affect the generalizability of the study's findings to the broader population of luxury brand consumers?
3. Were any statistical techniques, such as bootstrapping or parceling, employed to mitigate potential issues related to sample size?
4. How does the choice of test-train data split impact the performance and generalizability of the machine learning models used in the study?
5. Can you provide more details on why specific statistical techniques and machine learning models, such as Support Vector Machines (SVM), were chosen for this study?
6. What are the most significant findings of the study, particularly with regards to the influence of brand trust, prestige, uniqueness, and personalized recommendations on purchase intention?
7. What are the practical implications of the study's findings for luxury brand marketers, and how can they leverage Instagram to build brand trust and prestige?
8. What are the implications of using an SVM model for predicting adoption likelihood, and how does this contribute to our understanding of luxury brand marketing on Instagram?
9. How do the study's findings contribute to our understanding of luxury brand marketing on Instagram, and what are the practical implications for marketers seeking to optimize their Instagram strategies?
10. What avenues for future research are suggested by the study's findings, and how might these inform the development of more effective luxury brand marketing strategies on Instagram?

Is the work clearly and accurately presented and does it cite the current literature?

Yes

If applicable, is the statistical analysis and its interpretation appropriate?

Partly

Are all the source data underlying the results available to ensure full reproducibility?

Partly

Is the study design appropriate and is the work technically sound?

Yes

Are the conclusions drawn adequately supported by the results?

Yes

Are sufficient details of methods and analysis provided to allow replication by others?

Partly

Reviewer Expertise:

Statistics, Operations , Analytics and Market research

I confirm that I have read this submission and believe that I have an appropriate level of expertise to confirm that it is of an acceptable scientific standard, however I have significant reservations, as outlined above.

# Response

**Published:** 2025-10-09

**Authors:** Itam Urmila Jagadeeswari

**Affiliations:** Commerce, Manipal Academy of Higher Education, Manipal, Karnataka, India

We sincerely thank the reviewers for their thoughtful and constructive comments, which have greatly enhanced the quality and clarity of this manuscript. Detailed responses to each point raised in the review comments are provided below, addressing the suggestions and concerns individually.

 1. What are the implications of using a sample size of 205 for Structural Equation Modeling (SEM) analysis, and how might this impact the reliability of the findings?

**Response:** A sample size of 205 is adequate for path analysis, which was used in this study, as it falls within the commonly accepted range for SEM (Kline, 2015). However, for more complex models with many latent variables, a larger sample would be preferable. Our model was parsimonious, and all paths were statistically significant, indicating sufficient power for the tested relationships.

 2. How does the sample size of 205 affect the generalizability of the study's findings to the broader population of luxury brand consumers?

**Response:** While the sample size of 205 is reasonable for exploratory and model-building research, it may limit generalizability across diverse cultural or demographic contexts. The study focused on Indian Instagram users, and future replications with larger, cross-cultural samples are recommended to enhance external validity.

 3. Were any statistical techniques, such as bootstrapping or parceling, employed to mitigate potential issues related to sample size?

**Response:** Bootstrapping was not employed in this study, but the use of machine learning models like SVM and decision trees, which are robust to smaller samples, helped complement the statistical analyses. Future studies will incorporate resampling methods to further validate the stability of the estimates.

 4. How does the choice of test-train data split impact the performance and generalizability of the machine learning models used in the study?

**Response:** The models were evaluated using standard train-test splits (e.g., 70-30 or 80-20) to ensure performance metrics were not overfitted. This approach helps in assessing model generalizability to unseen data, as recommended in predictive analytics literature (Hastie et al., 2009).

 5. Can you provide more details on why specific statistical techniques and machine learning models, such as Support Vector Machines (SVM), were chosen for this study?

**Response:** SVM was chosen for its effectiveness in handling high-dimensional data and non-linear relationships, which are common in behavioral datasets (Cortes & Vapnik, 1995). Logistic regression was used for interpretability of psychological predictors, while decision trees offered transparent demographic segmentation.

 6. What are the most significant findings of the study, particularly with regards to the influence of brand trust, prestige, uniqueness, and personalized recommendations on purchase intention?

**Response:** Personalized recommendations and brand trust were the strongest predictors of purchase intention, followed by brand prestige. Brand uniqueness, while positive, was not significant, suggesting that differentiation alone may not drive adoption without emotional or relational reinforcement.

 7. What are the practical implications of the study's findings for luxury brand marketers, and how can they leverage Instagram to build brand trust and prestige?

**Response:** Marketers should prioritize personalized content, foster trust through authentic storytelling and influencer transparency, and leverage interactive formats (e.g., polls, Q&As) to build emotional connections. Psychographic segmentation using ML models can also enhance targeting efficiency.

 8. What are the implications of using an SVM model for predicting adoption likelihood, and how does this contribute to our understanding of luxury brand marketing on Instagram?

**Response:** The SVM model’s high accuracy (87%) demonstrates the value of psychological and social constructs in predicting adoption. This supports the use of AI-driven tools in luxury marketing for precise consumer profiling and campaign optimization.

 9. How do the study's findings contribute to our understanding of luxury brand marketing on Instagram, and what are the practical implications for marketers seeking to optimize their Instagram strategies?

**Response:** The study highlights that emotional and symbolic factor—more than surface-level engagement—drive luxury adoption. Marketers should focus on trust-building content, personalization, and emotional resonance rather than mere exposure metrics.

 10. What avenues for future research are suggested by the study's findings, and how might these inform the development of more effective luxury brand marketing strategies on Instagram?

**Response:** Future studies could explore longitudinal designs, cross-platform comparisons (e.g., Instagram vs. TikTok), integration of neuro-marketing tools, and advanced NLP for sentiment analysis to capture nuanced consumer emotions.

# Reviewer report: Reviewer response for version 1

**DOI:** 10.5256/f1000research.186997.r413334

**Published:** 2025-09-30

**Authors:** Anshu Rani

**Affiliations:** 1 REVA University, Bengaluru, Karnataka, India

Predicting Consumer Adoption of Luxury Products via Instagram Marketing: A Machine Learning Approach

 The purpose of this paper, "Predicting Consumer Adoption of Luxury Products via Instagram Marketing: A Machine Learning Approach," is to investigate the factors that influence the purchase of luxury products on Instagram. The study employs a mixed-methods approach to collect data from 205 participants in India. The study contributes on the idea that consumer adoption was not significantly influenced by superficial engagement metrics. Further, psychological and brand-related factors, including personalised recommendations, brand trust, and brand prestige, were acknowledged as robust and significant predictors of purchase intention. The study also highlights the nature of the content which is crucial for brand trust. High accuracy was demonstrated by machine learning models (Support Vector Machine and Decision Tree) in predicting the likelihood of an individual adopting a product based on psychological, social, and demographic factors. The sentiment analysis of qualitative feedback revealed that the majority of consumers had favourable sentiments towards Instagram luxury marketing; however, there were some concerns regarding the authenticity of influencers. The paper concludes that luxury marketing on Instagram should shift from a focus on sheer visibility to the cultivation of emotional connections, trust, and the provision of personalised, high-quality content.

 Comment: Revision

 This study conducts an investigation into the factors influencing luxury product adoption on Instagram, utilising a mixed-methods approach that integrates traditional statistical analysis with machine learning models.  The examination of Instagram marketing for luxury goods in the emerging market of India highlights a significant gap in existing literature and is highly pertinent to the field. Advanced analytical techniques involve the integration of machine learning models. The discovery that "surface-level" engagement metrics, such as time spent and story ads, lack significance, whereas psychological factors like trust, prestige, and personalisation are strong predictors, represents a crucial and actionable contribution.  This challenges traditional marketing metrics.

 Nonetheless, the study is considerably constrained by a significant limitation: The research is hindered by an absence of a unified theoretical framework, resulting in fragmented hypothesis testing. The theories are relevant; however, they are not cohesively integrated. It is essential to address these fundamental issues for the paper to achieve the standards of a high-quality indexing.

 The sample size (n=205) is insufficient for a machine learning approach and is exclusively from India, which complicates the applicability of the findings to other cases.

 The lack of clarity in the definition and examples of "luxury products" may indicate that individuals had in mind a variety of distinct categories of products.

 The study examines six distinct hypotheses utilising four varied analytical methods.  A comprehensive conceptual model illustrating the interrelations among the constructs of engagement, trust, purchase intention, and adoption is lacking.  The theoretical and empirical distinctions between H2 (Purchase Intention) and H4 (Adoption) remain ambiguous.  This results in the paper appearing as a collection of distinct mini-studies instead of a unified work.

 The results are presented clearly with helpful visualizations. However, the interpretation often oversteps the data by implying causality. The conclusions do tie the elements together but are framed with more certainty than the methodological design permits.

 Omitted Variable Bias: Unmeasured factors, such as general consumer materialism, brand affinity, and cultural trends, are likely to affect both the independent variables, including perceived brand trust, and the dependent variable, which is adoption intention.

 Discussion and Limitations: A specific subsection should be incorporated into the discussion and limitations sections to explicitly address the issue of endogeneity, clarifying that the results demonstrate predictive power rather than causal relationships.

 Future Research: The authors ought to propose methodologies for subsequent studies to determine causality, including longitudinal designs, field experiments, or the application of instrumental variables.

 Overall, I recommend a revision for the paper.

Is the work clearly and accurately presented and does it cite the current literature?

Yes

If applicable, is the statistical analysis and its interpretation appropriate?

Partly

Are all the source data underlying the results available to ensure full reproducibility?

Partly

Is the study design appropriate and is the work technically sound?

Yes

Are the conclusions drawn adequately supported by the results?

Yes

Are sufficient details of methods and analysis provided to allow replication by others?

Partly

Reviewer Expertise:

Online consumer behaviour, influencer marketing, UGC, quantititive method

I confirm that I have read this submission and believe that I have an appropriate level of expertise to confirm that it is of an acceptable scientific standard, however I have significant reservations, as outlined above.

# Response

**Published:** 2025-10-09

**Authors:** Itam Urmila Jagadeeswari

**Affiliations:** Commerce, Manipal Academy of Higher Education, Manipal, Karnataka, India

We sincerely thank the reviewers for their thoughtful and constructive comments, which have greatly enhanced the quality and clarity of this manuscript. Detailed responses to each point raised in the review comments are provided below, addressing the suggestions and concerns individually.

 This study conducts an investigation into the factors influencing luxury product adoption on Instagram, utilising a mixed-methods approach that integrates traditional statistical analysis with machine learning models.  The examination of Instagram marketing for luxury goods in the emerging market of India highlights a significant gap in existing literature and is highly pertinent to the field. Advanced analytical techniques involve the integration of machine learning models. The discovery that "surface-level" engagement metrics, such as time spent and story ads, lack significance, whereas psychological factors like trust, prestige, and personalisation are strong predictors, represents a crucial and actionable contribution.  This challenges traditional marketing metrics.

 Nonetheless, the study is considerably constrained by a significant limitation: The research is hindered by an absence of a unified theoretical framework, resulting in fragmented hypothesis testing. The theories are relevant; however, they are not cohesively integrated. It is essential to address these fundamental issues for the paper to achieve the standards of a high-quality indexing.

**Response:** We acknowledge the need for a more unified theoretical framework. While our study draws on the Theory of Planned Behavior, Social Identity Theory, and the Elaboration Likelihood Model, we have integrated these into a cohesive conceptual model. This model visually and theoretically links Instagram engagement, brand trust, purchase intention, sentiment, and adoption, clarifying how these constructs interrelate. This addresses the concern that the hypotheses appear fragmented and has been added to the literature review section as subsection 2.2 with the proposed conceptual framework.

 The sample size (n=205) is insufficient for a machine learning approach and is exclusively from India, which complicates the applicability of the findings to other cases.

**Response:** We recognize that a sample size of 205 may limit the generalizability of machine learning results. In the limitations section, we have explicitly stated that the sample size, while sufficient for initial insights, will be expanded in future research to improve model robustness and cross-cultural applicability. We also note that the study is focused on India, and future work will include diverse geographic contexts. (Please Refer page no – 30)

 The lack of clarity in the definition and examples of "luxury products" may indicate that individuals had in mind a variety of distinct categories of products.

**Response:** We have clarified the definition and examples of luxury products in the methodology section. The study focused on high-end fashion and accessories, such as luxury handbags, watches, apparel, and footwear, consistent with prior studies (e.g., Ko et al., 2019). This specificity has been explicitly stated to avoid ambiguity.

 The study examines six distinct hypotheses utilising four varied analytical methods.  A comprehensive conceptual model illustrating the interrelations among the constructs of engagement, trust, purchase intention, and adoption is lacking.  The theoretical and empirical distinctions between H2 (Purchase Intention) and H4 (Adoption) remain ambiguous.  This results in the paper appearing as a collection of distinct mini-studies instead of a unified work.

**Response:** A conceptual model diagram has been introduced to illustrate the relationships between engagement, psychological constructs, content types, sentiment, and adoption. This will help distinguish between purchase intention (H2) and adoption (H4), with adoption positioned as the ultimate behavioral outcome influenced by intention, trust, and other mediators.

 The results are presented clearly with helpful visualizations. However, the interpretation often oversteps the data by implying causality. The conclusions do tie the elements together but are framed with more certainty than the methodological design permits.

**Response:** We agree that causal claims are not supported by our cross-sectional design. The revised manuscript has more carefully been framed findings as predictive or associative, not causal. Language implying causation has been replaced with terms like "predictive association" or "influence," particularly in the results and discussion sections.

 Omitted Variable Bias: Unmeasured factors, such as general consumer materialism, brand affinity, and cultural trends, are likely to affect both the independent variables, including perceived brand trust, and the dependent variable, which is adoption intention.

**Response:** We acknowledge that unmeasured variables like materialism or brand affinity could influence results. This has explicitly been discussed as a limitation, and we have suggested that future studies include such constructs to better account for confounding effects.

 Discussion and Limitations: A specific subsection should be incorporated into the discussion and limitations sections to explicitly address the issue of endogeneity, clarifying that the results demonstrate predictive power rather than causal relationships.

**Response:** A new subsection has been added to the limitations addressing endogeneity. We have clarified that our models identify predictive relationships but cannot establish causality due to the research design and potential omitted variables.

 Future Research: The authors ought to propose methodologies for subsequent studies to determine causality, including longitudinal designs, field experiments, or the application of instrumental variables.

**Response:** We have expanded the future research section to include specific methods for establishing causality, such as longitudinal designs, field experiments, or instrumental variable approaches. This will provide a clearer pathway for subsequent studies to build on our findings.

 Thanks and Regards

 Authors

# Reviewer report: Reviewer response for version 1

**DOI:** 10.5256/f1000research.186997.r413335

**Published:** 2025-09-23

**Authors:** Singgih Santoso

**Affiliations:** 1 Universitas Kristen Duta Wacana Yogyakarta, Yogyakarta, Indonesia

This study has five objectives. However, in the discussion, there are six hypotheses. What is the connection between H6 and the previous five hypotheses?

 The five hypotheses appear to have no relation to one another. For example, the dependent variable in H1 is Consumer Adoption, while the dependent variable in H2 is Purchase Intention. What is the relationship between Consumer Adoption and Purchase Intention? Likewise, the dependent variable in H3 is Consumers’ Perception of Brand Trustworthiness. Furthermore, in H6, the term consumer sentiment suddenly appears. What is the connection between consumer sentiment and the variables from H1 to H5?

 It would be better if the author started by creating a diagram of the conceptual framework of this research and explained the roadmap for the development of the research hypotheses.

 The study uses luxury products as its objects, but the type and examples of luxury products used have not been explained in sufficient detail. Are they apparel products (bags, sandals, shoes), clothing, or something else? Since luxury products come in many forms.

 In figure 2, it is stated that H1 is not supported. Why does the analysis suddenly use logistic regression in H2 with different variables? What is the reason for using logistic regression to test H2 instead of multiple regression? Looking at the variables, the independent and dependent variables for H1 and H2 are completely different. What is being conveyed by using two different models for the same study?

 In the testing of H3, the analytical tool is path analysis (different from H1 and H2), with the dependent variable being Brand Trustworthiness. From figure 3, it can be seen that the engagement variable acts as the dependent variable, and not Brand Trustworthiness. Could you please explain this?

 as a review summary, we would like to ask why so many analysis tools are being used (Multiple Linear Regression, Logistic Regression, Path Analysis, Support Vector Machines (SVM), Decision Tree modeling, and Sentiment Analysis) to test the research questions. please explain the sequence of using these tools according to the existing roadmap

Is the work clearly and accurately presented and does it cite the current literature?

Yes

If applicable, is the statistical analysis and its interpretation appropriate?

Partly

Are all the source data underlying the results available to ensure full reproducibility?

Yes

Is the study design appropriate and is the work technically sound?

Yes

Are the conclusions drawn adequately supported by the results?

Yes

Are sufficient details of methods and analysis provided to allow replication by others?

Yes

Reviewer Expertise:

consumer behavior, digital marketing

I confirm that I have read this submission and believe that I have an appropriate level of expertise to confirm that it is of an acceptable scientific standard, however I have significant reservations, as outlined above.

# Response

**Published:** 2025-10-09

**Authors:** Itam Urmila Jagadeeswari

**Affiliations:** Commerce, Manipal Academy of Higher Education, Manipal, Karnataka, India

We sincerely thank the reviewers for their thoughtful and constructive comments, which have greatly enhanced the quality and clarity of this manuscript. Detailed responses to each point raised in the review comments are provided below, addressing the suggestions and concerns individually.

 1. This study has five objectives. However, in the discussion, six hypotheses are presented. What is the connection between H6 and the previous five hypotheses?

**Response:** H6 (positive consumer sentiment is associated with higher adoption) extends the quantitative findings by incorporating qualitative emotional insights. While the H1–H5 tests structural and behavioral relationships, H6 captures the affective dimension of consumer responses, enriching the understanding of adoption drivers. This aligns with research by Pang & Lee (2008) on the role of sentiment in shaping consumer behavior.

 2. The five hypotheses appear to have no relation to one another. For example, the dependent variable in H1 is consumer adoption, while the dependent variable in H2 is Purchase Intention. What is the relationship between Consumer Adoption and Purchase Intention? Likewise, the dependent variable in H3 is Consumers’ Perception of Brand Trustworthiness. Furthermore, in H6, the term consumer sentiment suddenly appears. What is the connection between consumer sentiment and the variables from H1 to H5?

**Response:** Consumer Adoption is the overarching outcome variable, while Purchase Intention (H2) and Brand Trustworthiness (H3) are mediating constructs that influence adoption. Sentiment (H6) acts as an affective precursor that shapes trust, intention, and adoption. This integrated view is supported by Bagozzi et al. (1999), who emphasize the role of emotion in decision-making.

 3.  It would be better if the author started by creating a diagram of the conceptual framework of this research and explained the roadmap for the development of the research hypotheses.

**Response:** We agree with the reviewer’s suggestion. A conceptual framework diagram will be included in the revised manuscript to visually illustrate the relationships between engagement metrics, psychological constructs, content types, sentiment, and adoption. This will clarify the research roadmap and hypothesis development.

 Figure 1 is attached to the manuscript. This box does not have a provision to paste. 

 The detailed theoretical framework and conceptual model is included in the sub-section 2.2 as suggested.

 4.  The study uses luxury products as its objects, but the type and examples of luxury products used have not been explained in sufficient detail. Are they apparel products (bags, sandals, shoes), clothing, or something else? Since luxury products come in many forms.

**Response:** The study focused on luxury fashion and accessories, including items such as handbags, watches, apparel, and footwear. This has been explicitly stated in the methodology section to enhance clarity, following the categorization used in prior studies (e.g., Ko et al., 2019). (Included in Page 13)

 5.  In figure 2, it is stated that H1 is not supported. Why does the analysis suddenly use logistic regression in H2 with different variables? What is the reason for using logistic regression to test H2 instead of multiple regression? Looking at the variables, the independent and dependent variables for H1 and H2 are completely different. What is being conveyed by using two different models for the same study?

**Response:** H1 used multiple linear regression to test the effect of continuous engagement metrics on adoption. H2 used logistic regression because purchase intention was measured as a binary outcome (likely/unlikely). This approach is methodologically appropriate and common in consumer behavior studies (e.g., Hair et al., 2014).

 6.  In the testing of H3, the analytical tool is path analysis (different from H1 and H2), with the dependent variable being Brand Trustworthiness. From figure 3, it can be seen that the engagement variable acts as the dependent variable, and not Brand Trustworthiness. Could you please explain this?

**Response:** The diagram (DAG) for path analysis clearly shows that different content types first affect consumer engagement, which then predicts brand trustworthiness. Engagement is included in the model as a mediating variable rather than the main dependent variable; the final outcome we measure and report is brand trustworthiness. This analytical strategy allows us to quantify both the direct effect of content types on trustworthiness and their indirect effect through enhanced engagement. Thus, while engagement appears as a central node in Figure 4, the path analysis fully retains brand trustworthiness as the dependent variable, fulfilling the hypothesis and model structure as described. Engagement’s placement helps clarify how content strategies exert layered influences rather than altering the outcome variable itself.

 7.  as a review summary, we would like to ask why so many analysis tools are being used (Multiple Linear Regression, Logistic Regression, Path Analysis, Support Vector Machines (SVM), Decision Tree modeling, and Sentiment Analysis) to test the research questions. please explain the sequence of using these tools according to the existing roadmap.

**Response:** The mixed-methods design required diverse tools to address different research questions: regression for direct effects, path analysis for mediation, machine learning for prediction, and sentiment analysis for qualitative insight. This multi-method approach is advocated by Shmueli & Koppius (2011) for comprehensive model validation and depth.

 Thanks & Regards

 Authors
